# Metabolomic Profiles of a Midge (*Procladius villosimanus*, Kieffer) Are Associated with Sediment Contamination in Urban Wetlands

**DOI:** 10.3390/metabo7040064

**Published:** 2017-12-18

**Authors:** Katherine J. Jeppe, Konstantinos A. Kouremenos, Kallie R. Townsend, Daniel F. MacMahon, David Sharley, Dedreia L. Tull, Ary A. Hoffmann, Vincent Pettigrove, Sara M. Long

**Affiliations:** 1 Centre for Aquatic Pollution Identification and Management (CAPIM), School of BioSciences, The University of Melbourne, Royal Pde, Parkville 3010, Australia; tok@unimelb.edu.au (K.R.T.); macmahon@unimelb.edu.au (D.F.M.); d.sharley@gmail.com (D.S.); vpet@unimelb.edu.au (V.P.); hoskins@unimelb.edu.au (S.M.L.); 2 Metabolomics Australia, Bio21 Molecular Science and Biotechnology Institute, 30 Flemington Road, Parkville 3052, Australia; konstantinos.kouremenos@unimelb.edu.au (K.A.K.); dedreia@unimelb.edu.au (D.L.T.); 3 Bio2lab Pty Ltd., 10/75 Main Hurstbridge Road, Diamond Creek 3089, Australia; 4 School of BioSciences, Bio21 Molecular Science and Biotechnology Institute, The University of Melbourne, 30 Flemington Road, Parkville 3052, Australia; ary@unimelb.edu.au

**Keywords:** biomonitoring, Chironomidae, environmental metabolomics, *Procladius villosimanus*, sediment contamination

## Abstract

Metabolomic techniques are powerful tools for investigating organism-environment interactions. Metabolite profiles have the potential to identify exposure or toxicity before populations are disrupted and can provide useful information for environmental assessment. However, under complex environmental scenarios, metabolomic responses to exposure can be distorted by background and/or organismal variation. In the current study, we use LC-MS (liquid chromatography-mass spectrometry) and GC-MS (gas chromatography-mass spectrometry) to measure metabolites of the midge *Procladius villosimanus* inhabiting 21 urban wetlands. These metabolites were tested against common sediment contaminants using random forest models and metabolite enrichment analysis. Sediment contaminant concentrations in the field correlated with several *P. villosimanus* metabolites despite natural environmental and organismal variation. Furthermore, enrichment analysis indicated that metabolite sets implicated in stress responses were enriched, pointing to specific cellular functions affected by exposure. Methionine metabolism, sugar metabolism and glycerolipid metabolism associated with total petroleum hydrocarbon and metal concentrations, while mitochondrial electron transport and urea cycle sets associated only with bifenthrin. These results demonstrate the potential for metabolomics approaches to provide useful information in field-based environmental assessments.

## 1. Introduction

Sediment contamination is an important factor affecting organisms in aquatic environments. Sediment provides food and shelter for many organisms but accumulates hydrophobic contaminants to concentrations an order of magnitude higher than water in the same system [1]. Changes in small metabolite profiles in organisms exposed to sediment contamination can represent early sub lethal responses to environmental exposure. Measuring metabolomic responses in exposed organisms can provide sensitive and rapid information on organism impacts [2,3,4]. An appropriate species to investigate these responses in field populations could be the non-biting midge *Procladius villosimanus. Procladius* is a genus from the family Chironomidae and represent a predominantly benthic group of midges, spending the majority of their lifecycle exposed to the sediment. *Procladius* are abundant, cosmopolitan and widespread in urban and agriculturally-impacted lake sediments [5,6,7]. Furthermore, *Procladius* display deformities after exposure to contaminated sediments [8], which could be associated with metabolomic changes.

There has been increased interest in the field of environmental metabolomics—the application of metabolomics to characterize the responses of organisms to their environment [9]. Environmental metabolomics can be used to investigate organism-environment interactions, helping to identify chemicals of concern within environmental mixtures, before a population is disrupted, as well as their modes of action [10,11,12]. Recently, it has been shown that metabolomics can indicate signatures predicting whole organism toxicity, which is a key aspect of environmental research [13,14]. It is possible, however, that toxic signals can be distorted by natural environmental or organismal variability. Differences in an organism’s developmental stage, sex, exposure time and physiochemical conditions can impact its metabolomic profile. To control for this variation, environmental metabolomic investigations have primarily been based on laboratory exposures of either lab or field-bred organisms [4,13,15,16] or field exposures of laboratory bred or translocated organisms [2,11,17,18,19,20]. Few studies exist on assessing metabolomic changes in resident organisms under field exposures [21,22]. Metabolomic studies in aquatic organisms have focused on water exposures, with only a few considering the influence of sediment exposure [23] and metabolism of sediment microbiota has also been investigated [12].

Untargeted metabolomic approaches (e.g., GC-MS, LC-MS (Hilic) and NMR profiling) are potentially useful for identifying organism-specific responses and discovering new molecular features [24,25,26]. If untargeted molecular features can be identified, they can also help discover specific and novel biomarkers of exposure and toxicity (e.g., [27]). In contrast, targeted metabolomic approaches (e.g., LC-MS profiling of amine-containing metabolites GC-MS profiling of free fatty acids) report on the abundance of groups of metabolites involved in specific cell responses [4,28]. These targeted techniques provide accurate detail on specific classes of metabolites but are limited as effects on unidentified or unanticipated biochemical pathways are not measured. The combination of these two approaches can therefore be powerful for environmental metabolomic investigations. Several amine-containing metabolites are involved in the transsulfuration pathway, which has been shown to be important in metal detoxification and antioxidant responses [4,16,29,30]. To target this class of metabolites this study included a targeted LC-MS of amine-containing metabolites. In addition to LC-MS, an untargeted GC-MS was included as an economical broad-spectrum measure of polar metabolites involved in a wide range of cellular functions including carbon cycle, ammonia cycle, citric acid cycle, carbohydrate and energy metabolism [4,31,32,33].

This study was designed to investigate the metabolite profile of *P. villosimanus* from independent sites with sediment containing different mixtures of contaminants. The aim was to establish if profiles of resident *P. villosimanus* could be linked to sediment contaminant exposure in the field and to investigate if metabolite profiles are associated with contaminants of concern in environmental samples.

## 2. Results

### 2.1. Sediment Contaminants

**Figure 1:**
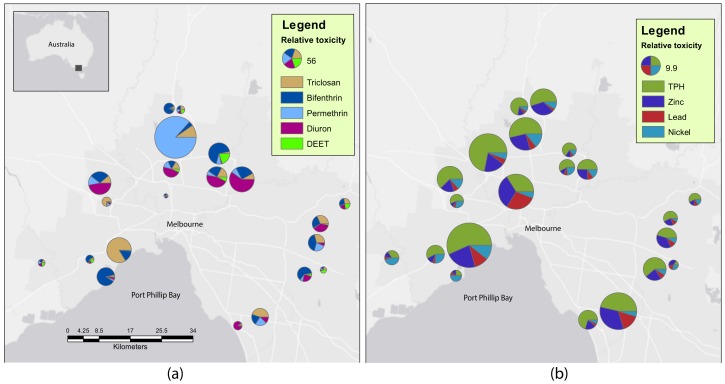
Location of 21 wetlands sampled in the Greater Melbourne Area. Including a visual representation of modelled (**a**) metal and hydrocarbon and (**b**) pesticide contamination at each location. Contaminant concentrations are displayed relative to ecological thresholds as described previously [34,35]. The size of each wheel represents the summed relative toxicity of presented contaminants for each map.

Sediment contamination was common in the sampled wetlands, with 6 pesticides occurring at more than 10% of sites (Table 1, Figure 1). The pesticides detected most frequently were bifenthrin and diethyltoluamide (DEET), which were above limit of detection (LOD) at 90.5% and 57.1% of the 21 sites, respectively. Zinc, lead, total petroleum hydrocarbon (TPH) and nickel contamination was also common in wetland sediments, occurring at 100% of sites and often at concentrations likely to cause ecological impacts. The detection frequency and potential ecological toxicity of contaminants are displayed in Table 1 and Figure 1. Generally, frequently detected contaminants that occurred at several sites above limit of reporting and below the low threshold (LOR < LT), above low threshold and below the high threshold (LT < HT) and above the high threshold (>HT) were selected for modelling. However, DEET, triclosan and diuron were selected for modelling based on their detection frequency and the variance between sites, although all concentrations were LOR < LT (Table 1, Figure 1).

### 2.2. Annotated Untargeted Metabolite Analysis of P. Villosimanus

For GC-MS, 177 metabolic features were included in random forest modelling. The identity of 87 of these features was confirmed with standards, a further 44 were identified using Fiehn and NIST11 databases and 46 remained unidentified metabolites. Data from GC-MS used for modelling are displayed in supplementary data (Table S1).

**Figure 2:**
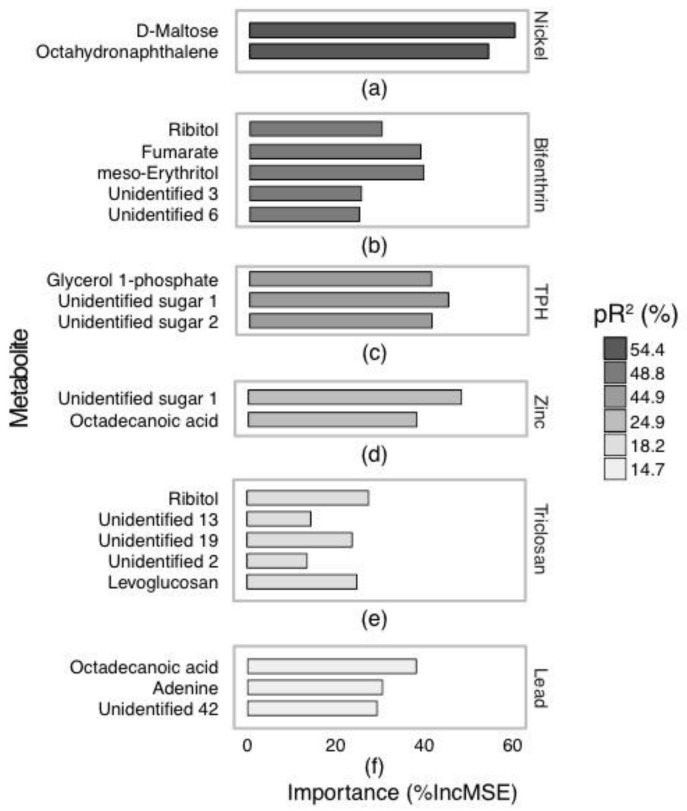
Relative importance of metabolites identified by GC-MS selected by model for association with (**a**) nickel, (**b**) bifenthrin, (**c**) total petroleum hydrocarbons (TPH), (**d**) zinc, (**e**) triclosan and (**f**) lead in *Procladius villosimanus* collected from 21 wetlands. Data were modelled with a random forest regression. Fills represent the goodness of fit of each model. Abbreviations: pR^2^, pseudo-R^2^; %IncMSE, percent increase in mean squared error.

**Figure 3:**
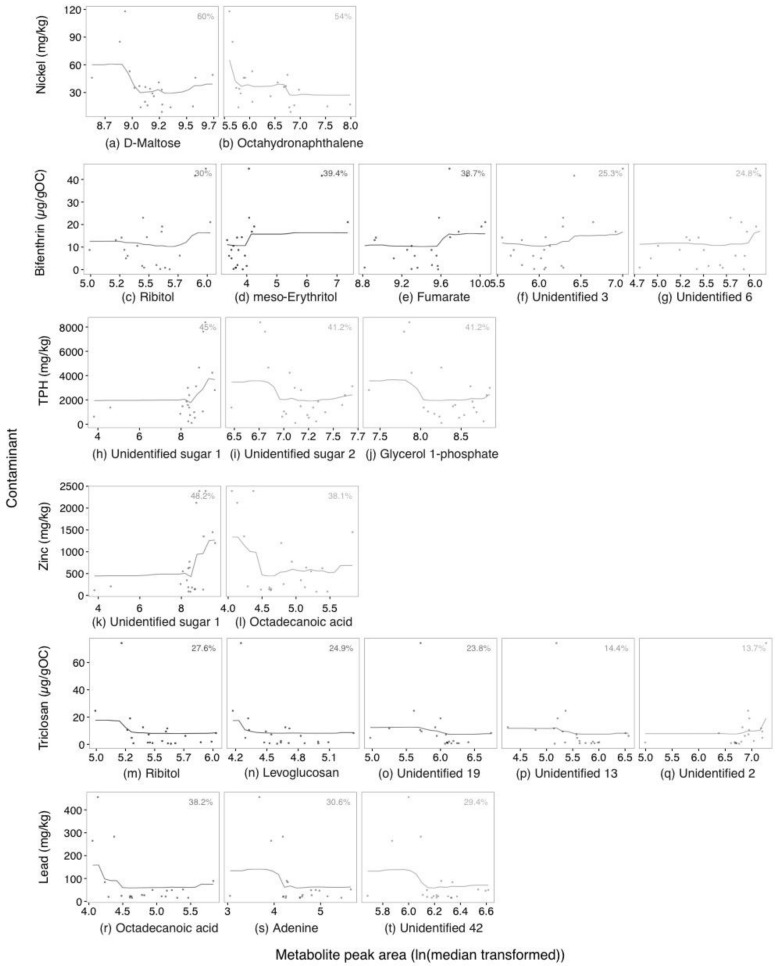
Results of random forest modelling, with partial dependence plots showing the association of *Procladius villosimanus* GC-MS annotated metabolites with nickel (**a**,**b**), bifenthrin (**c**–**g**), total petroleum hydrocarbons (TPH) (**h**–**j**), zinc (**k**,**l**), triclosan (**m**–**q**) and lead (**r**–**t**). Percent increase in mean squared error (%IncMSE) for each metabolite is indicated in the top corner of each panel and visualized with shading.

Model fit and importance of *P. villosimanus* untargeted GC-MS analysis are displayed in Figure 2 and Figure 3. Nickel produced the best fitting model for GC-MS data with 54.4% pR^2^ Figure 2a. D-maltose and octahydronaphthalene were considered important metabolites associated with nickel, with percent increase in mean squared error (%IncMSE) of 60 and 54%, respectively. When nickel concentrations were high D-maltose and octahydronaphthalene both decreased (Figure 3a,b).

The model for bifenthrin produced a pR^2^ of 48.8% and presented five important metabolites (Figure 2b). These metabolites were fumarate, meso-Erythritol, ribitol and unidentified metabolites 3 and 6 and had %IncMSE values between 24.8 and 39.4 (Figure 3c–g). All these metabolites increased with high bifenthrin.

The TPH model produced a pR^2^ of 44.9% and indicated that glycerol 1-phosphate and two unidentified sugars (1 (a monosaccharide) and 2 (a disaccharide)) were important (Figure 2c). Unidentified sugar 1 increased with high TPH (45%IncMSE) and glycerol 1-phosphate and unidentified sugar 2 decreased (both with 41.2%IncMSE) (Figure 3h–j).

Zinc produced a model with 24.9% pR^2^ indicating octadecanoic acid and unidentified sugar 1 as important metabolites (Figure 2d). Unidentified sugar 1 increased with increasing zinc and octadecanoic acid decreased (45%IncMSE) (Figure 3k–l).

Triclosan produced a model with 18.2% pR^2^ indicating five important metabolites (Figure 2e). These metabolites were ribitol, levoglucosan and three unidentified metabolites (2, 13 and 19) (27.6, 24.9, 13.7, 14.4 and 23.8%IncMSE, respectively). Unidentified metabolite 2 increased with high triclosan, ribitol and levoglucosan decreased, while unidentified metabolites 13 and 19 displayed no strong directional trend (Figure 3m–q).

Lead produced a model with 14.7% pR^2^ and showed three metabolites to be important: octadecanoic acid, adenine and unidentified metabolite 42 (38.2, 30.6, 29.4%IncMSE, respectively) (Figure 2f). Similar to nickel, these metabolites all decreased with increasing lead (Figure 3r–t).

Models for DEET, diuron and permethrin either could not be produced or produced a model with <10% pR^2^ so were not considered further.

### 2.3. Targeted Amine-Containing Metabolite Analysis of P. villosimanus 

**Figure 4:**
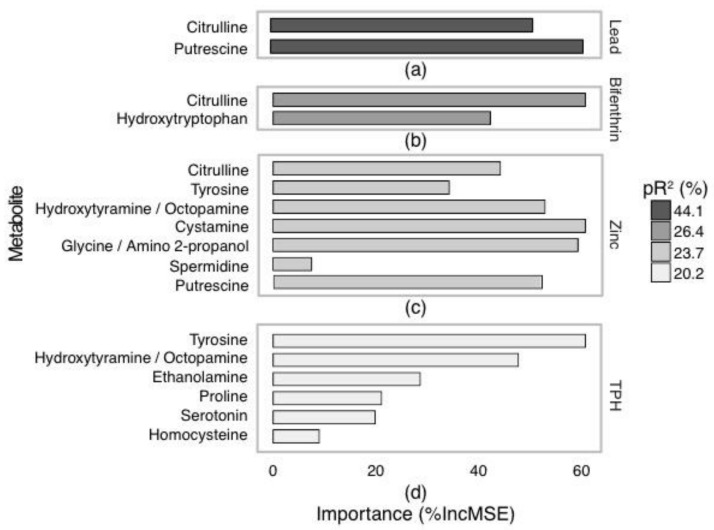
Relative importance of LC-MS amine metabolites selected by model for association with (**a**) lead, (**b**) bifenthrin, (**c**) zinc and (**d**) total petroleum hydrocarbons (TPH) in *Procladius villosimanus* collected from 21 wetlands. Data were modelled with a regression random forest model. Fills represent the goodness of fit of each model. Abbreviations: pR^2^, pseudo-R^2^; %IncMSE, percent increase in mean squared error.

**Figure 5:**
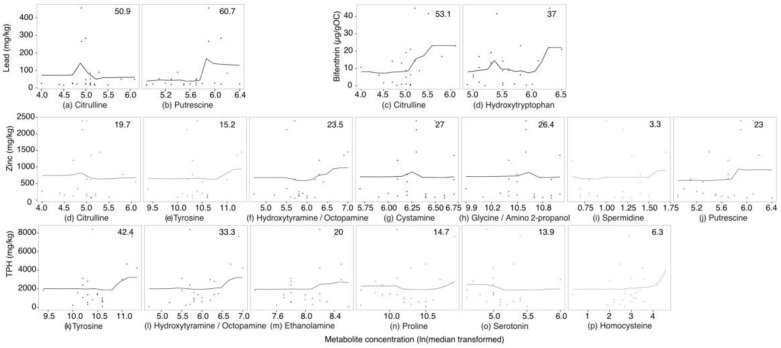
Results of random forest modelling, with partial dependence plots showing the association of *Procladius villosimanus* LC-MS metabolites with lead (**a**,**b**), bifenthrin (**c**,**d**), zinc (**d**–**j**) and total petroleum hydrocarbons (TPH) (**k**–**p**). Percent increase in mean squared error (%IncMSE) for each metabolite is indicated in the top corner of each panel and visualized with shading.

For targeted amine-containing metabolite analysis, 49 metabolites were detected. These included 27 amino acids, 7 neurotransmitters, 6 biogenic amines and 3 sulfur containing amines. Data from LC-MS used for modelling are displayed in Table S2. Random forest model fit and importance of *P. villosimanus* amine-containing metabolites are displayed in Figure 4. Lead achieved the best fitting model, with metabolite variation explaining 44.1% (pR^2^) of the variation (Figure 4a). Citrulline and putrescine were important metabolites associated with lead, with 50.9 and 60.7%IncMSE, respectively (Figure 5a,b). For citrulline, high lead clustered at the center of the chart with a metabolite concentration (ln(median transformed) around 5 (Figure 5a). Putrescine concentration tended to increase with increasing lead (Figure 5b).

Bifenthrin produced a model with 26.4% pR^2^ (Figure 4b), with Citrulline and hydroxytryptophan being important metabolites, representing 53.1 and 37%IncMSE, respectively (Figure 5c,d). While, an increase in citrulline was associated with high bifenthrin (Figure 5c), the relationship with hydroxytryptophan was less clear (Figure 5d).

Zinc produced a model with 23.7% pR^2^ and was associated with seven important metabolites (Figure 4c and Figure 5d–j). Of these metabolites, coeluted hydroxytyramine/octopamine, putrescine, tyrosine and spermidine tended to increase with increasing zinc presenting 23.5, 23, 15.2 and 3.3%IncMSE, respectively. Citrulline showed a similar relationship as it did with lead, with 19.7%IncMSE. Cystamine and the coeluted glycine/amino-2-propanol showed no directional trends (27 and 26.4%IncMSE, respectively).

The model for TPH presented a pR^2^ of 20.2% and indicated that six metabolites were important (Figure 4d and Figure 5k–p). Tyrosine, coeluted hydroxytyramine/octopamine and ethanolamine increased with high TPH presenting 42.4, 33.3 and 20%IncMSE respectively. Proline, serotonin and homocysteine were also important, with lower %IncMSE (14.7, 13.9 and 6.3, respectively), and no strong directional trends.

Models for nickel, triclosan, DEET, diuron and permethrin either could not be produced or produced a model with <10% pR^2^ so were not considered further.

### 2.4. Metabolite Set Enrichment Analysis of P. villosimanus

**Figure 6:**
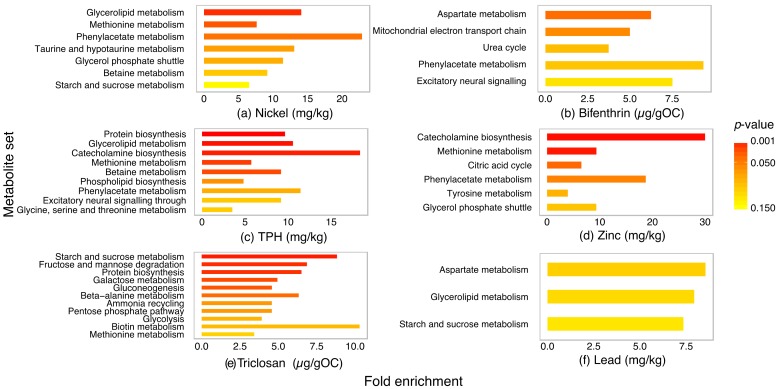
Results of an overrepresentation analysis (ORA) of important and marginally important metabolites (>3%IncMSE from random forest modelling) from GC-MS and LC-MS analysis of *Procladius villosimanus* associated with nickel (**a**), bifenthrin (**b**), total petroleum hydrocarbons (TPH) (**c**), zinc (**d**), triclosan (**e**) and lead (**f**). Color gradient indicates the *p*-value associated with each metabolite set.

The results of an overrepresentation analysis (ORA) of metabolites that were considered important and marginally important (>3%IncMSE) for each contaminant are displayed in Figure 6. As no *P. villosimanus* specific pathway databases are available, implications from ORA were based on databases for other species.

For nickel, enrichment was observed in glycerolipid, methionine and phenylacetate metabolism (Figure 6a). Metabolites associated with bifenthrin were involved in aspartate, mitochondria electron transport and urea cycle metabolite sets (Figure 6b). For TPH, enrichment was observed in protein biosynthesis, glycerolipid, catecholamine biosynthesis and methionine metabolism set (Figure 6c). For zinc, enrichment was observed in catecholamine biosynthesis, methionine metabolism and citric acid cycle (Figure 6d). Triclosan enriched metabolite sets associated with sugar metabolism and protein biosynthesis (Figure 6e). Lead associated with marginally significant enrichment of aspartate, glycerolipid and sugar metabolism (Figure 6f). Although the unidentified sugars could not be included in the enrichment analysis, TPH and zinc were also considered to associate with sugar metabolism.

## 3. Discussion

This study investigated the metabolite profiles of *P. villosimanus* from urban wetlands containing different mixtures of contaminants. The profiles of several metabolites associated with contaminants of concern in wetland sediments and ORA indicated enrichment of several metabolite sets. Furthermore, some metabolite sets that were enriched represented cellular responses to contaminant exposure.

The contaminant profiles of these wetland sediments are typical of moderately-contaminated urban wetlands. Aside from the general metal and hydrocarbon contamination, the widespread occurrence and elevated concentration of bifenthrin and triclosan are emerging concerns. Studies in Australia and elsewhere have reported environmental impacts of bifenthrin and other synthetic pyrethroids at similar concentrations to those observed here [36,37,38,39,40]. Triclosan, an antimicrobial used in many household products, has also recently been highlighted as a risk to environmental and human health [41,42]. Exposure to sediments with these contaminant profiles would likely cause metabolic stress responses in resident biota.

All sediment contaminants that could be modelled with random forests displayed directional associations with several metabolites. This is encouraging for the field of environmental metabolomics, where assessing resident organism responses under field exposures is desirable but may be distorted by natural environmental or organismal variability. It is likely that the contaminants that could not be modelled (DEET, diuron and permethrin) did not elicit a metabolic response in *P. villosimanus* that could be measured above natural environmental and organismal variation.

Metals and TPH shared several associated metabolites and enriched similar metabolite sets, namely, methionine metabolism, glycerolipid metabolism and sugar metabolism. These metabolite sets are of particular interest as they represent known exposure response pathways. Methionine metabolism leads to antioxidant responses and metal sequestration through production of metallothionein and glutathione S-transferase (among other products), which have previously been shown to respond to contaminant exposure [4,29,30,43,44].

Altered glycerolipid metabolism, through changes in glycerol-1-phosphate, palmitic acid and glycerol, could impact cellular membrane stabilization in these organisms. Particularly, the depletion of glycerol-1-phosphate could be responsible for changes in membrane structure and function. Previously, membrane metabolites have been reported to alter in fish exposed to mercury [23] and the glycerolipid metabolism specifically has been shown to respond to metal exposure [25]. Enrichment in glycerolipid and methionine metabolism metabolite sets provides strong evidence of exposure in *P. villosimanus.*

Several of the metabolites and sets that associated with bifenthrin were not associated with any other contaminants (mitochondrial electron transport and urea cycle). As a synthetic pyrethroid, bifenthrin disrupts ionic balances and osmoregulation in target organisms such as *P. villosimanus* [45]. The enriched metabolite sets were both altered through changes in fumarate and citrulline and researching these metabolites in combination could provide opportunities to develop bifenthrin exposure biomarkers. Triclosan produced a weak model for GC-MS, with the majority of metabolites being involved in sugar metabolism and no model for LC-MS data. As triclosan is an antimicrobial it is possible that the small responses observed here may be due to indirect effects on *P. villosimanus* or a response of microbes present in their gut.

Many of the metabolite sets enriched in *P. villosimanus* were involved in sugar metabolism. As accumulation of intracellular sugars, such as sucrose and carbohydrate reserves are often linked to cellular stress and reduced growth, these might be interesting biomarkers for assessing general environmental stress. Furthermore, these changes represent altered energy metabolism, which has often been found to alter due to environmental exposure [3,4,15,33].

The combination of targeted LC-MS and untargeted GC-MS provided useful information that can assist with field-based environmental monitoring. Although this combination of techniques is not comprehensive in the coverage of chemical space, they have provided useful information in several biochemical pathways of interest and have proved robust in an environmental setting.

To the authors knowledge this is the first research considering metabolomic assessments in resident midges exposed to contaminated field sediments, with results indicating that metabolic profiles of resident biota can provide useful information on field exposure. Several metabolomic profiles were linked to sediment contaminants and associated with contaminants of concern in these environmental samples. Furthermore, ORA results indicated that several metabolite sets were enriched in *P. villosimanus* and some of these sets associated with cellular responses to contaminant exposure. This research shows the potential for integrating metabolomics approaches into field-based environmental assessment.

## 4. Materials and Methods

### 4.1. Study Area and Procladius Collection

*Procladius villosimanus* individuals were collected from twenty-one constructed wetlands in the Greater Melbourne metropolitan Area (GMA) between the 26th of February and 7th of May 2015 (Figure 1). The latitudes and longitudes of these wetlands are listed in Table S3. For *P. villosimanus* collection, bare substrate was identified at each site and sampled for 2 min with a 250 µm dip net. Fourth instar *Procladius* spp. larvae were live picked from the sample on site until a minimum of 30 individuals were obtained or for 30 min. If after 30 min, 30 larvae had not been obtained the collection process was repeated for a maximum of three collection attempts. Resulting *Procladius* spp. were rinsed in deionized water, blot dried and snap frozen in dry ice for identification and metabolomic analysis. Once in the laboratory, the head capsule and ~2 mm of tissue was taken from each frozen *Procladius* spp. larvae for species identification and deformity assessment. Deformity assessment is not considered further in this article.

### 4.2. Sediment Chemical Analyses

Deposited sediments (top 2 cm) were collected and sieved on site through 63 µm nylon mesh [46]. Sediments were then allowed to settle in 10 L buckets, the overlying water decanted and remaining sediment stored in acid washed, acetone rinsed glass jars in the dark at 4 °C for chemical analysis. Sediments were analyzed for metals, pesticides, total organic carbon, petroleum hydrocarbons, polycyclic aromatic hydrocarbons (PAHs). Experimental details for sediment analysis have been published previously [34,35,36]. Briefly, University of Melbourne Chemistry Department analyzed the sediments for organic contaminants by GC-MS, including personal care products and selected pesticides. The QA/QC for GC-MS analysis is displayed in Table S4. Australian Laboratory Services (ALS) analyzed the sediments for total metals by ICP-AES (method: EG005T), total mercury by FIMS (method: EG035T), total petroleum hydrocarbons (C9-C36) (method: EP080/071), total organic carbon (TOC) (method: EP003) and moisture content (method: EA055). The QA/QC for ALS analysis is displayed in Table S5.

### 4.3. Species Identification of P. villosimanus

The head capsule of each larva was mounted in Hoyers medium and identified to species under a compound microscope and confirmed by DNA extraction and sequencing cytochrome oxidase I (COI) [47]. Larval DNA was extracted from ~2 mm of tissue using Chelex resin. Tissue samples were homogenized in 150 µL 5% Chelex resin (BioRad, Gladesville, Australia Cat No.: 1421253) with two 3 mm glass beads in a mixer mill at 25 Hz for 2 min. Samples were then centrifuged for 5 min at 2700× *g* and mixed with 5 µL proteinase K (Thermo Fisher Scientific Scoresby, Australia Cat No.: EO0491). Samples were incubated for 60 min at 65 °C followed by inactivation for 10 min at 90 °C. Samples were then centrifuged for 5 min at 2700× *g*, supernatant diluted 1/50 and stored at −20 °C until PCR amplification.

*Procladius* species were identified by amplifying and sequencing a 710 bp fragment of the COI gene using the universal primers LCO1490: 5′-ggtcaacaaatcataaagatattgg-3′ and HC02198: 5′-taaacttcagggtgaccaaaaaatca-3′ Folmer, et al. [48]. Each PCR reaction was 25 μL and contained the following components: 5 μL Chelex extracted DNA, 2.5 μL ThermoPol 10× buffer (New England Biolabs Ipswich, United States (NEB) Cat. No. B9004S), 2 min μL dNTP (25 mM), 0.2 μL Taq DNA polymerase (NEB Cat. No. M0273S), 1.25 μL each LCO1490 and HC02198 primers (10 μM each) and 12.8 μL of double deionized water. The qPCR program was: 3 min at 95 °C, followed by 35 cycles of 1 min at 95 °C, 1 min at 40 °C and 1 min 30 s at 72 °C, followed by a final extension step for 5 min at 72 °C. Amplification bands of correct size were confirmed with gel electrophoresis, using 2% agarose gels, stained with sybr safe (Thermo Fisher Scientific Cat No.: S33102). The PCR products were sequenced by Macrogen Inc. (Seoul, Korea) and resulting sequences were used in a BLAST search of nucleotide sequences from the family Chironomidae on Genbank [49] to identify the species. Larvae confirmed to be *P. villosimanus* were pooled into batches of 10 animals for metabolomic analysis, larvae of other species or with inconclusive DNA identification were excluded.

### 4.4. Metabolomic Analysis

#### 4.4.1. Metabolite Extraction

Depending on the abundance and size of identified fourth instar *P. villosimanus,* between 5 and 18 individuals were pooled for metabolomic analysis. This number of individuals provided enough biomass for between one and three replicate metabolite extractions per site (Table S3). Tissue for metabolite extraction was transferred into pre-weighed and pre-cooled 2 mL lysing tubes (containing 1.4 mm ceramic lysing beads (Bertin Technologies, Paris, France)) on dry ice and tissue weight was recorded. Metabolites were extracted using a modified Bligh-Dyer extraction method [50]. Samples were homogenized at 6800 rpm using a Precellys bead-mill attached to a Cryolys cooling unit (Bertin Technologies, Paris, France), pre-chilled with liquid nitrogen, at −10 °C in ice-cold 330 μL methanol and 110 μL deionized-distilled water containing internal standards (140 μM 13C5-15N-Valine and 14 μM 13C6-Sorbitol). Following homogenization, 110 μL ice-cold chloroform was added to each tube and the solutions were mixed thoroughly and metabolites further extracted at 2 °C for 15 min in a shaker. The tubes were then centrifuged at 14,000× *g* at 0 °C for 5 min. Supernatant was transferred to a fresh centrifuge tube and a further 220 μL deionized-distilled water was added to obtain a ratio of 3:3:1 (methanol:water:chloroform). This was mixed and centrifuged at 14,000× *g* at 0 °C for 5 min. The upper phase was collected into a fresh microcentrifuge tube and aliquots taken for LC-MS and GC-MS analysis. A pooled biological quality control (PBQC) was prepared by pooling 100 µL of the upper phase from all the *P. villosimanus* larvae extracts, mixed thoroughly and aliquoted into appropriate volumes for LC-MS (10 µL) and GC-MS (300 µL) and treated the same way as the samples prior to injection into the instrument.

#### 4.4.2. Annotated Untargeted Metabolite Analysis and Data Processing (GC-MS)

Untargeted metabolomics was performed using a GC-MS system (Agilent 7890A gas chromatograph coupled to an Agilent 5975C mass spectrometer (Santa Clara, CA, USA) with a Gerstel Autosampler (MPS 2 XL)). Around 300 μL of the extract was transferred into a GC-MS microvial insert, evaporated to dryness in a vacuum concentrator (Martin Christ, RVC 2-33, Osterode am Harz, Germany), 50 μL methanol was added and then dried again in a vacuum concentrator to ensure absolute dryness. The samples were derivatized online using a Gerstel MPS2 XL autosampler robot (Mülheim an der Ruhr, Germany). Each sample was treated with methoxyamine (Sigma Aldrich, St. Louis, MO, USA), in pyridine (Sigma Aldrich, St. Louis, MO, USA), (20 μL of 30 mg/mL, *w*/*v*) and the methoximated metabolites derivatized with *N*,*O*-bis-(trimethylsilyl)trifluoroacetamide (BSTFA) + 1% TMCS (20 μL, Pierce Technologies, Waltham, MA, USA). A 1 μL aliquot was injected splitless into the GC-MS for metabolite analysis. Gas chromatography was performed using a 30 m J & W Scientific VF-5 ms column (plus 10 m Eziguard pre-column Agilent Technologies, Santa Clara, CA, USA) with a 250 μm internal diameter and 0.25 μm film thickness. The injection inlet temperature was 250 °C, the GC-MS interface temperature 280 °C and the ion source temperature 250 °C. The carrier gas, helium, was used at a flow rate of 1 mL per minute. The GC oven temperature started at 35 °C (held for 2 min) and the temperature was ramped up by 25 °C per minute to 325 °C (held for 5 min). Mass spectra were recorded at 9.19 scans per second over an *m*/*z* range of 50 to 600.

Agilent GC-MS chemstation .D format files were converted to NetCDF format for analysis using the Metabolomics Australia GC-MS alignment and integration software, PyMS python toolkit [51]. The untargeted matrix output by PyMS was then ‘annotated’ using available authentic standards (using Agilent’s MassHunter Quantitative Analysis software) and combined, which in turn produced an ‘annotated untargeted matrix’. The level 1 metabolite standards initiative metabolites that were confirmed with authentic standards are listed in Table S1 [52,53]. Peaks that were detected were manually curated, validated and areas under the peak were calculated. Additional peaks which were not part of the authentic standards were then putatively annotated using the Fiehn (http://fiehnlab.ucdavis.edu/projects/fiehnlib) and NIST11 databases (https://www.nist.gov/srd). If ‘true’ peaks could not be putatively identified with databases, metabolites were included as numbered unidentified metabolites. Although a confident annotation could not be established, where possible unidentified sugars were annotated to class based on their ions and retention time (Table S6). After clean-up and annotation, the 224 metabolic features produced by analysis resulted in 177 ‘true’ peaks that could be included for modelling. This is a similar number of features compared with other untargeted GC-MS studies [31]. Table S6 contains the database matches for all PyMS detected metabolic features including retention time and quant ions.

#### 4.4.3. Targeted Amine-Containing Metabolomic Analysis and Data Processing (LC-MS)

Analysis of amine containing metabolites was performed by LC-MS according to Boughton, et al. [54]. Briefly, 10 μL of the extract was buffered by the addition of 70 μL borate buffer, pH 8.8 containing oxidizing and reducing agents 200 mM boric acid (Univar), 10 mM Tris(2-carboxyetgyl)-phosphine (TCEP, Sigma Aldrich), 1 mM Ascorbic acid (Sigma Aldrich), pH 8.8 (adjusted with 2 M NaOH) and 7.14 μM of ^13^C_3_-l-Alanine (Sigma Aldrich) as the internal standard. The reagent AQC (6-aminoquinolyl-*N*-hydroxysuccinimidyl carbamate, 20 μL, 10 mM stock in 100% acetonitrile, SAFC Sigma Aldrich) was added to the mixture and incubated for 10 min at 55 °C in a thermo mixer followed by centrifugation at 0 °C for AQC derivatization [54]. The supernatant was transferred to HPLC vials for LC-MS analysis. The samples (4 μL) were injected onto an Agilent 120 SB-C18 Poroshell 2.1 × 100 mm, 2.7 μm column and analyzed using an Agilent 1200-HPLC coupled to an Agilent 6460 Triple Quad MS (Agilent Technologies, Santa Clara, CA, USA). Amine containing compounds were quantified by dynamic multiple reaction monitoring mode (DMRM) using a fragmentor voltage range of 76–140 V, collision energy range of 9–25 V and collision gas (N_2_) at 10 L per minute. For most amine compounds, MRMs are as described in Boughton, Callahan, Silva, Bowne, Nahid, Rupasinghe, Tull, McConville, Bacic and Roessner [54]. Amine containing metabolites, MRMs and retention times are displayed in Table S7.

### 4.5. Statistical Analysis

All statistical analysis was performed in R (version 3.3.0 Supposedly Educational). For modelling, sediment contaminant concentrations were adjusted for limits of detection (LOD). Values below the LODs were reported as 50% of the LOD and trace detects were reported as 75% the LOD and all pesticide concentrations were then normalized to µg per gram organic carbon (µg/gOC). Contaminants were selected as response variables for modelling if they occurred at more than 10% and displayed reasonable variance between sites. Metals and hydrocarbons were included if more than two sites had concentrations exceeding the high threshold (>HT) (Table 1). Pesticides were included if more than five sites were above the low threshold (>LT) (Table 1). Thresholds were derived from local [55] and international [56,57] acute and chronic thresholds. Low thresholds were based on chronic effects such as observable effect concentration (NOEC), while high thresholds were based on acute effects such as median lethal concentrations (EC50). More detail on the derivation of these thresholds has been published [34].

For GC-MS, 281 metabolite features were identified; once noise was removed and peak derivatives summed these were reduced to 177 features to include in modelling. Metabolite data from available replicates of each site were averaged before analysis. Concentration (targeted amine-containing metabolites LC-MS) and area under the curve (untargeted GC-MS) data were median log-transformed to account for variation in sample weight and heteroscedasticity of metabolomic data. Where targeted amine-containing metabolites (LC-MS) eluted together a single measurement was included in analysis under the names of both metabolites.

Variable selection using bootstrap aggregation was used to identify the most important metabolites predicting sediment contaminants [58]. A random forest regression model was then constructed for each contaminant against the selected important targeted amine-containing (LC-MS) or untargeted (GC-MS) metabolites and results were visualized with partial dependency plots. Random forest models are non-linear multiple regression techniques based on decision trees. This technique allows for non-linear responses and estimate the relative importance of each predictor variable on the response variable. Random forest models were selected because they have previously been shown to be useful for complex environmental data [34,59,60,61].

The goodness of fit for each random forest model provides a pseudo-R^2^ (pR^2^), indicating the percent variance explained by the model. Partial dependency plots for each model indicate the predicted values for the response variable (contaminant concentration) for a given value of important predictor variables (metabolites).

An overrepresentation analysis (ORA) was then performed with MetaboAnalyst [62,63]. For this analysis, marginally important metabolites (>3%IncMSE) from unrestricted GC-MS and LC-MS random forest models were included. The ORA tests all metabolites with database identifiers against KEGG pathways for overrepresentation [64]. No *P. villosimanus* specific pathway databases are available so implications from ORA were based on databases or other species [62,63].

## Figures and Tables

**Table 1:** Contaminants that were frequently detected in wetland sediments. Entries provide the detection frequency (%) and the number of sites (out of 21) where each contaminant was below Limit of Reporting (<LOR), above LOR and below the Low Threshold (LOR < LT), above Low Threshold and below the High Threshold (LT < HT) and above the High Threshold (>HT). Thresholds have been defined previously [34,35]. Contaminants selected for random forest models are indicated with *.

Chemical Class	Contaminant	Detection Frequency (%)	<LOR	LOR < LT	LT < HT	>HT
Hydrocarbon	TPH *	100	0	2	1	18
Metal	Zinc *	100	0	8	3	10
Metal	Lead *	100	0	14	4	3
Metal	Nickel *	100	0	7	11	3
Metal	Copper	100	0	12	8	1
Metal	Chromium	100	0	19	2	0
Insecticide	Bifenthrin *	90.5	2	0	1	18
Metalloid	Arsenic	76.2	5	14	2	0
Insecticide	DEET *	57.1	9	12	0	0
Antimicrobial	Triclosan *	52.4	10	11	0	0
Herbicide	Diuron *	47.6	11	10	0	0
Metal	Mercury	42.9	12	4	4	1
Metal	Cadmium	33.3	14	2	4	1
Insecticide	Permethrin *	33.3	14	1	5	1
Fungicide	Pyrimethanil	19	17	4	0	0
Metal	Silver	9.5	19	0	0	2
Metalloid	Antimony	9.5	19	0	2	0
Fungicide	Trifloxystrobin	9.5	19	2	0	0
Insecticide	Fenamiphos	4.8	20	0	1	0
Herbicide	Prometryn	4.8	20	0	1	0

## Supplementary Materials

The following are available online at www.mdpi.com/2218-1989/7/4/64/s1, Table S1: GC-MS metabolite features included in random forest modelling, Table S2: LC-MS metabolite features included in random forest modelling, Table S3: Location of 21 wetlands sampled for this study, Table S4: Quality assurance and control for organics analysis by GC-MS in wetland sediments, Table S5: Quality assurance and control for metal and hydrocarbon analysis. Table S6: Database identification of GC-MS metabolites. Table S7: Amine containing metabolites measured with LC-MS.
