# Supplementary material for: Metabolomic Profiles of a Midge (Procladius villosimanus, Kieffer) Are Associated with Sediment Contamination in Urban Wetlands
Source: Metabolites. 2017 Dec 18;7(4):64. doi: 10.3390/metabo7040064 (PMC5746744; doi:10.3390/metabo7040064)
Supplement: Supplementary file 1 [file metabolites-07-00064-s001.pdf]

Metabolomic Profiles of a Midge (*Procladius villosimanus*, Kieffer) are Associated with Sediment Contamination in Urban Wetlands

Katherine J. Jeppe, Konstantinos A. Kouremenos, Kallie Townsend, Daniel MacMahon, David Sharley, Dedreia L. Tull, Ary A. Hoffmann, Vincent Pettigrove and Sara M. Long

Table S1. Metabolite features from GC-MS included in random forest modelling. Data are median and log transformed and listed under SiteID. Metabolites that have metabolite standards initiative level 1 identification confirmed with standards with authentic standards using Agilent's MassHunter Quantitative Analysis software are indicated in bold [1,2].

	SiteID																				
Metabolite	25	301	302	306	312	314	326	395	465	698	848	931	936	937	940	945	954	960	963	985	990
1,2,5-Oxadiazole-3,4-dicarboxamide	7.1	5.9	8.2	7.7	8.0	6.1	8.3	6.5	6.0	6.3	6.1	6.4	7.5	6.4	7.4	8.3	7.5	8.2	6.6	6.5	6.7
17-beta-Acetoxyandrostane	6.2	6.1	6.7	6.5	6.2	7.0	6.2	6.1	6.4	6.6	6.4	6.5	6.2	6.3	6.0	5.9	6.1	6.7	6.9	6.5	6.4
1H-Cycloprop[e]azulen-4-ol	6.5	6.5	7.2	7.1	6.2	7.5	6.5	6.0	7.0	6.4	6.2	6.5	6.4	7.4	6.2	6.3	7.0	6.7	5.8	6.0	5.4
2-Anthracenecarboxylic_acid,	6.9	7.2	6.6	5.1	7.2	5.7	6.8	7.1	6.7	6.8	7.3	6.7	6.7	5.9	6.5	6.6	6.6	6.4	7.3	6.8	7.1
2-Methylcortisol	7.3	6.7	6.8	6.9	7.2	6.7	6.6	7.2	6.4	6.4	6.6	6.5	6.1	6.2	6.1	6.4	7.1	6.7	7.1	6.5	6.5
2-Piperidone	10.1	10.4	10.2	9.9	10.3	10.4	10.2	10.4	10.2	10.4	10.6	10.1	10.9	9.9	10.8	10.7	9.9	10.5	10.1	10.8	10.6
2,6-di-t-butyl-4-(bromomethyl)phenol	6.7	6.6	7.0	6.9	6.3	6.8	6.6	6.6	6.6	6.4	6.6	6.6	6.7	7.1	6.4	6.4	6.7	6.5	6.2	6.3	6.3
3-Bromo-N-(3,5-dichlorophenyl)benzamide	5.7	5.3	5.6	5.1	5.2	5.8	5.2	5.4	5.6	5.1	5.9	5.4	5.1	5.4	5.0	3.9	5.6	5.2	5.2	4.9	5.2
3-phosphoglyceric_acid	8.4	6.6	7.8	7.2	7.6	7.3	6.8	7.2	6.7	7.8	6.0	7.3	6.5	7.7	7.0	7.1	7.2	7.9	7.9	7.5	7.4
3,4-dihydroxyphenylacetic_acid	7.6	8.1	8.2	7.9	8.6	7.7	8.0	8.3	8.0	8.2	8.2	8.1	8.1	7.4	8.1	8.2	7.9	8.2	8.5	8.4	8.4
5-Hydroxytryptophol	6.1	7.2	6.7	7.1	6.3	7.8	6.5	6.6	7.4	7.1	7.9	6.8	7.5	6.6	7.0	7.6	6.6	7.7	6.4	7.2	6.6
5.alpha.-androstane	6.7	6.6	6.3	6.5	6.7	6.4	6.6	6.7	6.7	6.3	6.9	6.5	7.4	7.0	7.0	7.4	6.7	6.9	6.3	7.0	6.9
9H-Purine	6.7	6.5	5.6	6.0	5.7	6.1	6.1	5.9	6.9	6.7	6.1	7.2	6.8	5.5	6.0	6.7	6.3	6.7	6.0	6.9	6.8
adenosine-5-monophosphate	7.9	7.4	7.8	7.2	7.4	7.8	7.2	7.5	7.7	7.3	7.2	7.6	7.2	7.8	6.8	6.6	7.2	7.6	8.1	7.5	7.4
alpha-D-Galactopyranoside	5.7	5.7	6.6	5.9	6.2	6.2	5.9	5.8	6.3	5.9	6.2	6.2	5.7	5.9	5.9	6.6	6.2	5.7	6.0	5.6	5.6

	SiteID																				
Metabolite	25	301	302	306	312	314	326	395	465	698	848	931	936	937	940	945	954	960	963	985	990
Androstan-17-ol,_acetate	6.4	6.6	6.8	6.5	6.0	6.8	6.7	6.7	6.7	6.5	7.2	6.7	6.6	6.8	6.6	7.3	6.4	6.2	5.6	6.4	6.1
Benzenebutanoic_acid	7.0	6.9	7.2	5.9	6.8	6.9	6.4	6.4	6.8	6.7	6.2	6.9	6.1	6.2	6.3	6.4	6.5	6.8	7.2	6.9	7.1
Citrulline_2	8.6	8.8	8.7	8.6	8.8	9.0	8.5	8.9	8.7	8.9	9.0	8.7	9.1	8.5	9.1	9.0	8.5	9.1	8.8	9.3	9.1
Cortisone	7.4	7.4	6.9	6.9	6.9	6.9	7.3	7.9	7.1	7.2	7.6	6.9	7.7	6.9	7.3	7.5	6.7	6.8	6.9	6.9	7.1
D-Erythrose-4-phosphate	7.1	7.1	7.2	7.2	8.0	7.1	7.5	8.0	7.4	7.2	8.3	7.2	8.4	7.0	8.7	8.7	7.3	6.5	7.0	7.6	7.9
DL-Leucine	6.9	7.0	7.2	6.8	6.8	6.8	7.1	6.8	6.9	7.3	7.7	7.0	6.9	7.6	7.2	7.3	6.9	7.5	7.0	7.4	7.4
Glucopyranose	9.7	9.0	9.8	9.1	9.7	8.6	8.1	9.6	8.1	7.9	8.0	8.9	9.1	8.4	8.1	7.7	9.7	9.3	9.5	7.5	8.3
Glycerol	8.5	8.2	8.3	8.3	8.2	8.3	8.0	8.1	10.2	9.8	8.6	8.4	8.0	8.5	8.0	8.2	7.9	7.8	8.3	8.1	8.0
Glycerol_1-phosphate	8.2	8.4	8.0	8.1	7.8	8.6	8.2	7.4	8.5	8.5	8.7	8.3	8.6	8.3	8.8	8.9	7.9	8.4	7.9	8.8	8.7
Inosine	9.0	8.5	9.1	8.7	8.5	8.4	8.4	8.1	8.7	8.2	8.1	8.6	7.9	9.5	8.0	7.7	9.2	8.2	8.4	8.1	8.6
Isopropyl_beta-D-1-thiogalactopyranoside	6.7	7.1	7.8	7.3	7.5	7.7	7.1	7.0	7.7	7.4	7.3	7.4	6.8	7.5	6.9	7.0	7.0	7.4	8.0	7.4	7.2
L-glutamic_acid	11.1	11.6	11.7	10.8	11.6	11.2	11.7	11.5	11.3	11.4	11.4	11.1	11.5	11.5	11.7	11.9	11.2	11.3	11.5	11.6	11.5
L-Glutamine_3	9.2	9.1	9.1	8.8	9.0	9.1	9.0	9.5	8.9	9.5	9.7	8.9	9.1	8.8	9.0	9.1	8.6	9.8	9.1	9.9	10.0
L-Lysine	10.8	11.1	11.3	10.5	10.8	11.0	11.3	11.1	11.0	11.2	11.1	11.0	11.6	11.1	11.6	11.1	10.9	11.3	10.9	11.6	11.3
L-Serine	9.0	9.3	9.4	9.0	9.2	8.3	9.5	9.2	9.2	9.3	9.4	9.1	9.6	9.3	9.3	9.4	9.0	9.3	9.0	9.7	9.4
Maltose	6.3	6.2	6.3	6.3	5.2	6.0	6.0	6.6	6.3	5.8	6.8	6.3	6.7	4.8	6.3	6.4	6.2	5.4	5.0	5.7	5.7
methyl-beta-D-Galactopyranoside	8.5	8.0	8.3	8.7	7.1	8.5	7.8	8.1	8.5	8.5	8.2	8.1	8.6	8.3	7.9	7.8	8.3	8.9	7.2	8.1	8.2
N-acetyl-D-mannosamine	7.5	7.9	7.4	6.2	7.8	7.0	7.7	6.2	8.3	7.6	7.1	6.7	6.9	7.8	7.5	6.9	7.5	6.4	8.3	6.4	6.6
N-acetyl-L-histidine	7.2	7.0	8.1	7.1	7.2	7.5	7.0	7.0	6.9	6.8	6.2	7.3	6.3	7.5	5.0	5.8	7.0	7.0	7.8	6.9	6.7
Octahydronaphthalene	5.8	5.8	5.9	7.0	5.9	6.6	6.8	5.7	5.8	6.0	6.9	6.7	6.8	7.5	6.7	6.7	5.6	8.0	5.7	6.4	6.1
Palmitic_acid	7.5	7.9	7.0	7.0	7.0	7.1	7.6	7.6	8.2	7.3	7.5	7.1	7.9	7.2	8.2	8.2	6.8	7.6	8.1	7.8	7.7
Pantothenic_acid	6.6	7.9	6.8	7.0	6.9	7.7	6.4	6.6	7.0	7.6	6.8	7.4	7.9	7.7	7.6	7.1	6.5	7.3	7.4	8.1	7.4
Pentanedioic_acid	7.3	7.5	8.1	8.0	7.5	8.4	8.2	8.2	7.1	8.0	7.9	7.7	7.5	7.5	8.4	8.6	7.8	8.6	7.5	8.6	8.9
Phenylalanine	9.0	9.3	9.3	9.0	9.0	8.8	9.4	9.2	9.0	8.9	9.0	9.2	9.6	9.2	9.3	9.2	9.1	9.0	9.0	9.7	9.4

	SiteID																				
Metabolite	25	301	302	306	312	314	326	395	465	698	848	931	936	937	940	945	954	960	963	985	990
Phosphoric_acid	9.2	9.8	9.8	9.0	9.6	9.9	9.9	9.5	9.8	9.9	10.0	9.5	10.1	9.7	10.6	10.9	9.2	10.0	9.6	10.1	10.0
Stearate	6.9	7.5	6.6	6.6	6.6	6.5	7.1	7.1	7.6	6.9	6.9	6.7	7.3	6.9	7.7	7.7	6.2	7.4	8.1	7.4	7.2
Unidentified_sugar_1_(monosaccharide)	3.8	8.1	4.6	9.0	9.1	8.7	8.0	9.6	8.4	8.7	8.6	8.9	8.3	8.5	8.3	8.3	9.2	8.3	9.5	8.4	8.4
Unidentified_sugar_2_(disaccharide)	7.0	7.2	6.5	7.0	6.8	7.7	7.0	7.2	7.2	7.3	7.4	6.8	7.3	7.1	7.3	7.1	6.8	7.6	7.1	7.6	7.3
Unidentified_sugar_3_(disaccharide)	6.9	7.1	7.7	7.7	6.9	7.9	7.0	6.8	7.5	7.2	7.1	7.0	7.1	8.0	6.7	6.8	7.4	7.3	6.4	6.5	6.6
Unidentified_1	7.6	7.5	7.9	7.8	7.8	8.3	7.6	7.6	7.7	7.6	8.1	7.8	7.9	7.6	7.2	5.8	7.4	7.8	7.9	8.0	7.8
Unidentified_10	5.6	6.9	6.9	6.3	7.4	5.6	6.7	7.0	6.2	6.3	6.8	6.8	6.2	5.9	6.8	7.2	6.5	6.0	7.5	6.7	6.8
Unidentified_11	6.6	6.1	5.7	9.4	9.1	7.0	4.1	8.7	5.8	6.3	8.7	5.6	6.1	7.2	7.3	8.1	7.4	9.8	4.1	9.9	6.1
Unidentified_12	6.7	8.4	8.1	7.4	8.5	8.0	8.0	8.1	8.1	8.4	7.5	8.1	8.4	7.8	8.7	8.6	7.1	8.3	8.5	8.8	8.7
Unidentified_13	5.6	5.6	5.6	4.8	6.6	5.2	4.3	5.6	5.3	6.0	5.9	5.2	5.9	5.4	6.0	6.5	5.2	5.7	5.4	5.9	5.1
Unidentified_14	5.7	5.7	6.4	6.3	5.9	6.8	6.6	6.5	6.1	6.3	5.8	6.1	6.4	6.0	4.8	5.5	5.8	6.4	6.4	6.1	6.6
Unidentified_15	7.8	6.5	6.7	7.4	6.9	6.0	5.9	6.6	6.0	6.1	6.0	6.5	6.6	6.7	6.3	5.6	7.3	6.3	6.2	6.4	6.0
Unidentified_16	4.5	4.9	4.8	4.4	3.9	5.9	5.0	5.4	3.7	4.1	5.9	4.9	7.7	3.7	6.7	5.0	7.0	5.9	5.2	6.4	6.1
Unidentified_17	8.2	8.1	8.3	7.9	8.0	8.5	8.5	8.3	8.4	8.8	9.1	8.4	9.2	7.9	9.1	8.8	7.8	8.6	8.4	8.9	8.8
Unidentified_18	6.7	7.1	7.0	7.1	7.3	7.4	7.1	6.9	7.4	7.4	7.3	6.9	7.4	7.0	7.5	6.9	7.0	6.6	6.8	7.6	7.0
Unidentified_19	5.0	6.1	6.3	5.7	5.9	6.1	5.9	6.2	5.7	6.1	6.1	6.0	6.2	6.1	6.4	6.7	5.7	6.1	5.6	6.2	5.2
Unidentified_2	7.2	6.9	6.7	6.9	6.9	6.8	6.8	7.0	7.2	6.7	6.8	7.0	6.7	5.0	6.7	6.4	7.3	6.6	6.9	6.7	7.2
Unidentified_20	9.4	9.6	10.0	9.3	9.0	10.5	10.4	8.8	10.0	9.6	9.4	9.8	9.5	8.5	8.5	8.9	8.2	8.8	9.7	9.2	10.3
Unidentified_21	7.3	7.3	7.7	7.4	7.6	7.7	7.2	7.4	7.6	7.5	7.8	7.4	7.0	7.3	7.1	6.9	7.5	7.0	7.3	7.0	7.2
Unidentified_22	5.3	5.6	5.7	5.6	5.9	6.0	5.7	5.4	5.9	5.9	5.8	5.5	5.8	5.6	5.9	5.5	5.5	5.2	5.4	6.1	5.6
Unidentified_23	8.3	8.1	8.1	8.1	8.3	8.5	7.7	8.3	8.2	8.0	8.3	8.1	7.6	7.5	7.5	7.0	8.2	8.0	7.8	7.6	8.0
Unidentified_24	6.3	6.3	7.1	6.3	6.8	6.8	6.5	6.6	6.9	6.6	6.8	6.9	5.9	6.2	6.2	5.9	6.8	6.1	6.6	6.2	6.3
Unidentified_25	5.6	5.9	5.7	5.6	5.8	6.2	5.9	5.4	6.3	6.0	6.2	5.8	5.8	5.6	5.5	5.0	5.8	5.6	5.4	6.1	6.0
Unidentified_26	7.2	6.3	6.9	6.9	6.9	6.6	6.3	6.9	6.2	6.4	6.6	6.5	6.3	6.5	6.3	5.2	6.9	6.4	6.7	6.1	5.4
Unidentified_27	7.9	7.2	7.5	7.5	7.6	7.3	7.0	7.7	7.2	7.2	7.4	7.0	7.0	7.4	7.6	7.8	7.5	7.0	7.4	7.5	6.9

	SiteID																				
Metabolite	25	301	302	306	312	314	326	395	465	698	848	931	936	937	940	945	954	960	963	985	990
Unidentified_28	7.0	7.3	7.0	6.9	7.1	7.3	6.9	7.3	7.1	7.3	7.5	7.1	7.5	6.9	7.7	7.7	6.9	7.4	7.3	7.6	7.5
Unidentified_29	8.0	6.7	6.7	8.0	7.2	6.0	3.8	6.6	5.2	5.1	5.2	7.4	5.8	6.0	5.0	4.4	7.1	7.5	7.1	5.3	6.1
Unidentified_3	6.0	6.3	5.9	5.6	5.7	6.3	6.1	5.8	5.9	6.3	7.0	6.1	6.1	6.1	6.4	6.6	5.8	6.1	5.7	6.3	6.9
Unidentified_30	4.9	5.2	4.9	5.0	5.0	5.4	5.2	5.3	4.9	5.1	5.4	4.9	5.7	4.8	5.5	6.0	5.4	5.3	6.2	5.8	5.4
Unidentified_31	7.4	7.6	6.9	6.4	5.8	6.1	7.0	6.8	7.1	7.8	7.4	7.1	7.2	6.8	8.7	6.7	6.9	8.5	6.0	8.0	7.2
Unidentified_32	9.3	9.3	10.1	9.4	9.5	9.6	9.1	9.5	9.4	8.9	9.3	9.5	8.7	9.8	8.4	8.7	9.3	9.4	9.6	8.9	9.1
Unidentified_33	7.0	6.7	7.4	7.4	7.1	6.9	7.3	7.5	6.7	7.1	7.3	6.7	7.4	6.4	7.7	7.5	7.3	7.4	7.1	8.0	7.4
Unidentified_34	5.9	5.6	5.7	5.8	7.3	6.1	7.6	5.5	6.0	7.3	5.8	6.4	6.9	7.8	6.7	7.2	5.5	7.2	5.3	6.8	6.1
Unidentified_35	7.5	7.6	7.8	7.6	7.9	7.9	7.6	7.5	7.9	7.7	7.7	7.6	6.9	7.6	6.9	7.9	7.7	7.4	7.9	7.4	8.0
Unidentified_36	6.4	6.4	6.4	6.2	6.1	6.6	6.1	6.0	6.1	5.7	5.5	6.2	5.0	6.1	5.1	5.0	6.2	5.9	7.2	5.9	5.8
Unidentified_37	7.2	6.9	7.6	7.2	7.3	7.1	7.6	6.7	7.0	7.0	6.5	7.2	6.7	7.5	6.6	6.7	7.5	7.0	7.5	6.8	7.3
Unidentified_38	6.9	6.1	5.6	6.6	6.7	6.5	6.2	6.3	7.0	6.9	6.5	6.2	6.9	6.2	6.9	6.8	6.6	5.5	6.2	6.8	6.0
Unidentified_39	8.3	8.1	8.9	8.9	7.8	9.2	8.0	7.6	8.7	8.0	7.7	8.1	7.9	9.2	7.3	7.0	8.7	8.1	7.5	7.0	7.0
Unidentified_4	8.7	8.9	9.0	8.5	8.3	9.3	9.0	8.2	8.7	8.9	9.5	8.9	8.4	9.0	8.5	8.8	8.5	9.1	8.8	8.9	9.5
Unidentified_40	7.2	7.6	7.0	6.6	7.4	7.3	7.3	7.5	7.3	7.2	7.7	7.0	7.7	6.7	7.8	8.1	7.1	6.7	6.8	7.4	7.6
Unidentified_41	9.2	9.9	9.7	9.2	9.8	9.3	9.9	9.5	9.7	9.9	10.0	9.4	10.2	10.1	10.6	10.7	9.2	10.0	9.6	10.2	9.8
Unidentified_42	6.2	6.1	6.1	5.7	6.3	6.0	6.2	6.1	6.2	6.3	6.2	6.1	6.3	6.2	6.3	6.5	5.9	6.4	6.3	6.6	6.6
Unidentified_43	5.9	6.2	6.3	6.0	5.7	6.1	6.0	5.7	6.1	5.5	6.2	5.8	5.6	6.9	5.8	5.2	6.0	5.6	5.8	5.8	5.6
Unidentified_5	9.6	8.7	9.8	9.3	9.2	9.0	9.8	10.0	9.2	9.5	9.0	9.0	9.8	9.2	9.4	9.6	9.5	10.1	9.3	9.6	9.8
Unidentified_6	4.9	5.8	5.9	5.4	5.8	5.7	5.0	5.7	5.4	5.6	6.1	5.2	5.9	4.8	6.1	5.9	5.3	5.3	5.5	6.1	6.0
Unidentified_7	8.3	8.6	6.4	7.9	8.2	7.1	7.0	8.8	7.8	8.2	8.7	8.1	9.3	6.7	9.4	9.3	8.0	8.3	7.6	8.8	8.8
Unidentified_8	7.5	7.5	7.7	7.7	6.8	7.7	7.5	7.7	7.6	7.3	8.0	7.7	7.9	7.8	7.5	8.0	7.5	7.0	6.0	7.1	7.0
Unidentified_9	7.9	8.6	8.7	7.9	8.6	8.5	8.8	8.5	8.6	8.7	8.6	8.2	9.1	8.6	9.2	9.4	8.1	8.6	8.4	9.0	8.7
Uracil	7.4	7.7	7.4	7.0	7.3	7.0	7.0	7.3	7.1	6.8	7.3	7.1	7.7	7.6	7.7	7.9	7.5	6.8	7.2	7.2	7.3
Uric_acid	9.3	9.9	9.8	9.2	9.7	9.7	10.1	9.7	9.8	9.9	10.0	9.6	10.3	9.9	10.5	10.9	9.3	10.0	9.6	10.1	10.0

	SiteID																				
Metabolite	25	301	302	306	312	314	326	395	465	698	848	931	936	937	940	945	954	960	963	985	990
Xanthurenic_acid	8.0	7.9	7.9	7.3	8.1	8.0	7.8	7.6	8.0	7.8	7.9	7.9	7.7	8.1	7.8	7.7	7.9	7.6	7.9	7.9	8.3
**Levoglucosan**	4.3	4.5	4.5	4.3	4.8	4.6	4.7	4.6	4.5	4.6	5.0	4.3	4.9	4.5	5.1	5.3	4.3	4.7	4.2	5.0	4.7
**6-Phosphogluconic_Acid**	1.7	2.0	1.9	1.8	1.5	1.9	2.0	1.9	2.1	1.7	2.4	1.8	2.1	2.0	1.8	2.7	1.6	1.5	0.7	1.5	1.4
**Adenine**	4.2	4.8	4.2	3.1	4.3	3.7	4.8	4.6	4.2	4.6	4.6	4.2	5.1	4.3	5.4	5.6	3.9	4.6	4.3	5.0	4.9
**Adenosine-5-monophosphate**	2.6	1.9	3.0	2.5	2.2	3.2	2.7	1.9	2.6	2.9	2.4	2.5	1.7	2.2	0.6	0.5	2.4	3.1	3.1	2.1	2.2
**Benzoic_Acid**	4.3	4.8	4.6	4.2	4.4	4.4	4.7	4.7	4.6	4.6	4.9	4.5	5.3	4.4	5.3	6.1	4.3	4.5	5.0	4.8	5.0
**beta-Alanine**	6.3	5.6	6.1	6.0	6.0	6.5	5.9	6.1	6.2	6.1	6.3	6.4	6.5	6.2	6.6	6.9	6.0	6.4	6.4	6.8	6.5
**Caffeic_acid**	1.1	1.0	2.4	2.4	2.7	2.2	1.3	1.8	1.4	1.3	2.4	1.2	2.6	1.2	1.9	2.0	1.2	3.1	1.1	2.7	2.0
**cis-Aconitic_acid**	3.3	3.2	3.0	4.1	3.7	2.5	2.8	3.2	2.3	2.7	3.1	3.2	2.4	1.9	2.6	4.2	3.4	5.4	3.5	4.8	3.6
**Citric_acid**	5.5	5.9	5.7	5.6	5.4	4.8	5.0	5.8	4.9	5.4	5.5	5.9	5.1	3.8	5.4	6.0	5.5	5.7	6.2	5.4	5.9
**D-(-)-3-Phosphoglyceric_acid**	6.1	4.2	5.5	4.9	5.3	5.0	4.5	4.9	4.5	5.5	4.0	5.0	4.2	5.5	4.9	4.9	4.9	5.6	5.6	5.2	5.1
**D-Erythrose**	1.1	1.9	1.8	1.5	1.6	1.6	1.8	2.2	1.7	1.4	2.1	1.7	1.9	2.0	2.0	2.6	1.5	1.7	1.3	1.3	1.5
**D-Fructose**	5.8	5.9	5.8	5.6	6.0	5.8	6.2	5.7	5.7	6.0	6.3	5.6	5.8	6.0	6.3	6.4	5.5	5.8	5.8	6.3	6.0
**D-Galactose-6-phosphate**	5.0	4.6	5.0	4.8	5.3	4.9	4.7	4.8	4.9	4.8	4.7	4.8	4.3	4.9	4.6	4.6	4.7	4.6	4.7	4.7	4.8
**D-Gluconic_Acid**	4.1	4.1	4.7	4.4	4.9	3.8	4.1	4.2	3.7	3.6	4.4	4.3	3.5	3.8	4.0	5.1	4.3	4.0	5.6	3.7	4.4
**D-Gluconic_Acid-Delta-Lactone**	4.0	4.4	4.5	4.0	4.1	4.1	4.5	4.3	4.2	4.0	4.8	4.2	4.7	4.4	4.3	5.4	4.1	4.3	4.0	4.1	4.0
**D-Glucosamine**	2.4	2.0	3.1	3.3	3.6	3.5	4.1	3.1	3.2	3.3	3.6	2.3	4.3	2.0	4.3	4.9	2.6	2.1	2.4	4.2	3.6
**D-Maltose**	9.1	9.2	9.6	9.3	8.6	9.2	9.3	9.0	9.2	9.0	9.1	9.1	9.4	9.6	9.1	9.7	8.9	9.3	8.9	9.2	9.1
**D-Myo-Inositol-1-phosphate**	5.0	5.1	5.1	4.8	4.9	5.3	5.2	5.0	5.2	5.2	5.1	5.0	5.4	4.7	5.4	5.1	4.7	5.2	5.2	5.3	5.5
**D-Ribose**	3.3	4.2	4.0	3.7	4.2	3.7	3.9	4.1	3.8	3.9	4.4	3.6	4.5	4.1	4.7	4.8	3.7	3.9	3.5	4.2	4.2
**D-Ribose-5-phosphate**	2.4	3.0	2.2	3.1	3.9	2.7	3.9	3.8	3.6	3.4	4.2	2.7	3.2	1.6	3.5	3.9	3.5	3.8	4.2	3.8	1.9
**D-Ribulose-5-phosphate**	2.9	2.9	2.5	2.8	3.3	2.7	3.2	2.9	2.7	3.2	3.2	2.6	3.7	2.5	3.8	2.4	2.6	3.0	2.4	3.5	3.1
**D-Talose_**	8.9	9.4	9.5	8.7	9.4	9.3	9.6	9.3	9.4	9.5	9.6	9.2	9.8	9.5	10.0	10.2	9.0	9.5	9.3	9.7	9.5
**D-Turanose**	8.0	8.4	8.6	7.8	7.8	8.4	8.7	8.2	8.4	8.7	8.5	8.3	9.0	8.7	8.7	9.4	7.9	8.8	8.3	8.9	8.6
**D-Xylose**	4.9	5.2	5.4	5.1	5.3	5.1	5.5	5.4	5.1	5.4	5.1	4.9	5.5	5.1	5.6	5.3	5.2	5.6	5.4	5.5	5.5

	SiteID																				
Metabolite	25	301	302	306	312	314	326	395	465	698	848	931	936	937	940	945	954	960	963	985	990
**DL-Homoserine**	4.2	4.4	4.0	4.2	4.5	4.4	4.1	4.1	3.4	4.5	4.6	3.8	4.6	4.3	4.7	5.0	3.9	4.7	3.8	5.2	4.0
**Fructose-6-phosphate**	5.0	4.7	6.0	5.6	5.8	5.7	6.2	5.3	5.2	6.1	5.5	5.2	5.5	6.2	5.7	5.8	4.9	6.0	5.1	6.4	5.2
**Fumarate**	9.1	9.6	9.3	8.9	9.6	9.4	9.3	9.5	9.3	9.6	9.7	9.3	9.6	8.8	9.9	10.1	8.9	9.7	9.5	10.0	9.8
**gamma-Aminobutyric_acid_(GABA)**	6.1	7.1	6.6	6.2	6.0	6.5	6.6	7.6	6.1	6.5	6.2	6.0	7.3	6.3	7.1	6.9	6.1	6.5	6.2	6.9	6.6
**Glucose**	8.9	9.4	9.4	8.8	9.4	9.2	9.5	9.3	9.4	9.4	9.5	9.1	9.7	9.5	9.8	10.1	8.9	9.5	9.3	9.6	9.4
**Glucose-6-phosphate**	6.4	6.2	7.3	6.8	7.1	6.9	7.6	6.7	6.4	7.3	6.9	6.5	7.1	7.4	7.2	7.6	6.3	7.3	6.5	7.7	6.6
**Glutamic_acid**	8.9	9.4	9.4	8.8	9.4	9.0	9.4	9.3	9.1	9.1	9.2	9.2	9.3	9.3	9.5	9.6	9.0	9.1	9.3	9.4	9.3
**Glutamine**	7.0	6.9	6.9	6.5	6.8	6.8	6.8	7.3	6.6	7.2	7.5	6.7	6.9	6.5	6.8	6.9	6.4	7.6	6.8	7.7	7.8
**Glyceric_acid**	4.9	4.8	4.5	4.1	4.8	4.0	4.6	4.6	4.2	4.5	4.6	4.3	4.7	5.1	5.1	5.6	4.5	4.3	4.5	4.9	5.1
**Glycerol-2-phosphate**	4.1	4.4	4.4	4.2	4.4	4.1	4.4	4.3	3.8	4.1	4.4	4.1	4.3	3.9	4.5	4.6	4.1	4.1	4.3	4.4	4.3
**Glycine**	8.8	9.0	9.2	9.1	9.0	9.0	9.0	9.3	8.9	9.1	9.1	9.0	9.4	9.3	9.2	9.5	9.1	9.5	9.0	9.4	9.1
**Hexadecanoic_acid**	5.3	5.7	4.8	4.7	4.7	4.8	5.4	5.3	6.0	5.1	5.3	4.9	5.7	4.9	6.0	6.0	4.5	5.4	5.9	5.5	5.5
**Hypoxanthine**	7.4	7.8	7.0	6.9	7.3	7.1	7.1	7.5	7.3	7.0	7.6	7.2	7.8	7.0	7.9	8.1	7.5	6.7	6.6	7.3	7.6
**Isocitric_Acid**	4.8	5.2	5.0	4.7	4.8	4.0	4.3	5.1	4.2	4.8	4.8	5.1	4.5	3.3	4.8	5.3	4.7	5.0	5.3	4.7	5.2
**L-Alanine**	9.2	9.6	9.7	9.5	9.9	9.0	9.6	9.5	9.2	9.8	9.4	9.3	10.0	10.0	10.3	10.5	9.5	10.0	9.3	10.4	9.3
**L-Asparagine**	8.2	8.5	8.8	8.5	8.6	8.5	8.8	8.7	8.5	8.7	8.5	8.3	8.9	8.5	8.9	8.7	8.5	9.0	8.8	8.8	8.8
**L-Aspartic_acid**	7.9	8.5	8.0	8.4	7.7	7.7	7.5	7.9	7.7	7.4	8.1	7.8	8.4	8.2	7.9	7.5	8.3	7.7	8.4	8.7	8.3
**L-Cystine**	0.1	0.2	0.7	0.8	0.3	0.8	2.0	0.6	0.5	0.6	0.8	0.4	2.1	0.4	1.7	2.1	0.1	1.5	0.6	1.1	1.4
**L-Histidine**	9.5	9.6	10.0	9.5	9.5	9.9	9.5	9.6	9.9	9.6	9.7	9.8	9.4	9.6	9.2	9.3	9.5	9.6	10.0	9.7	9.7
**L-Isoleucine**	9.0	9.2	9.3	8.9	9.1	8.7	9.3	9.2	9.1	9.1	9.2	9.0	9.6	8.9	9.5	9.6	8.9	9.1	9.2	9.7	9.3
**L-Leucine**	8.2	8.5	8.5	8.0	8.5	8.0	9.2	8.6	8.4	8.4	8.5	8.3	9.1	8.8	9.2	9.9	8.2	8.4	8.2	9.2	8.8
**L-Malic_acid**	8.2	8.7	8.6	8.0	8.6	8.5	8.7	8.6	8.5	8.7	8.8	8.4	9.0	8.5	9.3	9.5	8.2	8.8	8.6	9.0	8.8
**L-Methionine**	8.1	8.2	8.1	8.1	7.7	8.0	8.2	8.1	8.0	7.9	8.6	8.1	8.5	8.1	8.3	7.4	8.0	8.1	7.8	8.5	8.4
**L-Ornithine**	7.5	7.9	7.9	8.0	7.4	7.7	8.2	7.9	7.7	7.7	8.0	8.1	8.1	7.8	7.9	7.5	8.0	8.3	7.8	8.2	8.2
**L-Phenylalanine**	8.2	8.5	8.5	8.2	8.2	8.0	8.6	8.3	8.2	8.1	8.2	8.4	8.7	8.4	8.4	8.3	8.3	8.2	8.2	8.9	8.5

	SiteID																				
Metabolite	25	301	302	306	312	314	326	395	465	698	848	931	936	937	940	945	954	960	963	985	990
**L-Proline**	9.3	9.6	9.8	8.9	9.7	9.4	9.9	9.5	9.7	9.8	9.8	9.5	10.0	9.6	10.0	10.4	9.3	9.6	9.6	9.9	9.7
**L-Threonine**	8.5	8.7	8.8	8.3	8.6	8.5	8.9	8.7	8.7	8.6	8.8	8.7	8.9	8.7	9.0	9.3	8.5	8.7	8.7	9.0	9.0
**L-Tryptophan**	8.9	9.0	9.1	8.5	8.7	8.6	9.0	8.8	8.7	8.7	8.6	9.0	8.9	8.8	8.6	8.8	8.7	8.6	9.2	9.3	9.1
**L-Tyrosine**	9.7	10.2	10.3	9.6	10.3	9.9	10.4	10.1	10.1	10.2	10.3	10.1	10.3	10.1	10.6	11.0	9.8	10.1	10.4	10.5	10.2
**L-Valine**	9.3	9.7	9.8	9.2	9.6	9.4	9.8	9.6	9.6	9.8	9.8	9.5	10.0	9.7	10.0	10.4	9.3	9.8	9.6	10.1	9.8
**Lactic_Acid**	8.5	9.8	9.9	8.0	9.3	9.1	10.0	9.5	9.5	9.8	9.5	9.4	10.0	9.6	10.1	10.6	8.9	9.9	9.6	9.8	9.5
**Lysine**	8.6	8.9	9.1	8.8	8.6	8.9	9.2	9.0	8.9	9.0	9.0	8.8	9.4	9.0	9.4	9.0	8.8	9.1	8.7	9.4	9.1
**Maleic_acid**	3.8	4.1	4.1	3.6	4.2	3.8	4.0	4.1	4.1	4.1	4.4	3.9	4.5	3.7	4.7	4.9	3.7	4.0	3.9	4.4	4.2
**Malonic_acid**	1.7	1.7	1.5	1.5	1.4	1.6	1.5	1.7	1.4	1.6	1.5	1.6	2.0	1.4	2.1	2.4	1.7	1.6	2.2	2.2	2.0
**Maltotriose**	6.3	6.6	8.2	7.7	6.3	7.7	7.2	5.9	7.3	6.6	6.5	7.1	6.3	8.5	3.9	6.0	6.4	7.4	6.3	5.9	5.8
**Mannitol**	8.3	8.8	8.9	8.2	8.8	8.1	9.0	8.7	8.8	8.9	9.0	8.6	9.1	8.9	9.2	9.4	8.4	8.9	8.7	9.0	8.9
**meso-Erythritol**	3.5	4.1	3.4	3.7	3.9	3.6	3.6	3.7	3.6	4.0	4.1	3.5	3.9	3.6	6.5	7.4	3.3	3.8	3.7	4.2	4.2
**Myo-Inositol**	6.3	6.4	6.6	6.1	6.7	6.4	6.3	6.6	6.1	6.4	6.7	6.3	6.8	6.2	6.8	6.7	6.6	6.4	6.5	6.9	6.5
**N-acetyl-L-Glutamic_acid**	4.0	4.1	4.6	3.8	4.4	4.5	4.0	4.0	4.1	4.4	4.1	4.1	3.9	4.4	4.0	4.1	3.7	4.4	4.5	4.6	4.1
**Norleucine**	4.1	4.3	4.5	4.3	4.2	4.5	4.5	4.7	4.3	4.4	4.4	4.4	4.8	4.6	4.5	4.9	4.3	4.7	4.3	4.7	4.5
**O-Phosphoethanolomine**	1.6	0.6	0.5	0.8	0.7	1.1	0.5	0.2	0.6	0.5	0.6	1.0	0.6	0.9	0.6	0.9	0.7	0.6	0.2	0.4	0.4
**Octadecanoic_acid**	4.6	5.2	4.3	4.5	4.2	4.1	4.8	4.8	5.3	4.6	4.6	4.4	5.1	4.6	5.5	5.4	4.1	5.1	5.8	5.1	4.9
**Oxalic_acid**	1.7	2.2	2.4	1.8	2.5	2.2	2.5	2.1	2.2	2.5	2.4	2.0	2.9	2.4	2.9	3.2	1.8	2.4	2.0	2.7	2.4
**Phosphoenolpyruvate**	4.9	2.6	3.8	2.9	3.3	2.9	2.6	3.4	2.7	2.8	2.2	3.3	2.3	4.1	2.9	2.2	3.5	3.2	3.5	3.1	3.2
**Proline,_trans-4-hydroxyl-L-**	7.4	7.7	7.6	7.3	7.4	7.5	7.7	7.7	7.5	7.5	7.8	7.5	7.9	7.5	7.8	8.0	7.3	7.8	7.6	7.7	7.8
**Putrescine**	7.1	7.3	7.7	7.6	7.4	7.7	7.5	8.0	7.4	6.8	7.1	7.7	7.8	7.6	7.4	7.3	7.6	8.3	7.5	8.0	7.9
**Pyridoxine**	1.8	2.2	1.6	0.9	1.7	1.3	2.3	1.9	1.6	2.0	2.1	1.8	2.6	1.8	3.2	2.3	1.6	2.2	1.6	2.5	2.3
**Pyroglutamic_acid**	9.7	10.0	10.0	9.6	9.8	9.8	10.1	9.9	9.9	9.9	10.1	9.9	10.3	9.9	10.2	10.6	9.6	10.2	9.9	10.1	10.1
**Ribitol**	5.3	5.4	5.3	5.3	5.8	5.6	5.4	5.6	5.6	5.4	6.0	5.3	5.7	5.5	5.9	6.0	5.2	5.5	5.0	5.6	5.6
**scyllo-Inositol**	5.5	5.9	4.7	4.6	4.5	5.5	6.5	5.1	4.3	5.3	6.1	6.6	6.4	5.1	6.0	6.4	5.4	5.8	5.0	6.4	7.1

	SiteID																				
Metabolite	25	301	302	306	312	314	326	395	465	698	848	931	936	937	940	945	954	960	963	985	990
**Serine**	9.0	9.2	9.3	8.9	9.1	9.0	9.4	9.1	9.1	9.2	9.3	9.0	9.5	9.2	9.2	9.4	8.9	9.2	8.9	9.7	9.3
**Sorbitol**	8.2	8.8	8.8	8.0	8.7	8.6	9.0	8.7	8.7	8.9	8.9	8.4	9.1	8.9	9.2	9.4	8.3	8.9	8.5	9.0	8.7
**Spermidine**	3.6	3.3	3.4	3.4	3.6	3.6	3.7	3.5	3.4	3.8	4.0	3.7	4.3	3.1	4.2	4.5	3.1	3.7	3.7	4.2	3.7
**Succinic_acid**	6.7	7.6	7.2	7.4	7.6	7.4	7.8	7.2	7.1	7.8	7.2	7.1	8.4	6.5	8.4	7.5	7.2	6.6	7.7	8.4	6.9
**Sucrose**	3.4	4.5	4.3	3.0	3.0	4.2	3.3	4.0	3.6	4.0	3.8	3.4	3.5	4.3	4.4	3.9	4.5	3.6	3.5	4.7	3.9
**Tartaric_acid**	2.4	2.8	2.6	1.9	2.7	3.2	1.8	2.2	2.3	2.6	3.3	2.8	2.5	2.2	2.8	2.9	2.1	3.1	3.0	3.1	3.3
**Taurine**	2.8	3.1	3.9	3.4	4.0	2.6	2.8	3.7	2.5	2.9	1.9	3.1	2.7	1.8	3.3	1.9	3.1	2.8	3.2	2.6	3.8
**Tetracosanoic_acid**	2.7	3.5	3.1	1.4	3.5	2.5	3.6	3.7	3.1	3.4	3.9	2.3	3.0	2.6	2.5	0.9	2.6	3.1	3.9	3.4	2.9
**Tetradecanoic_acid**	4.0	4.0	3.0	2.8	3.4	3.3	3.9	3.7	4.4	3.5	3.9	3.2	4.2	3.2	4.2	4.5	2.8	3.5	3.7	3.9	4.0
**Trehalose**	8.3	8.5	8.4	8.3	8.7	8.3	9.4	9.1	8.8	9.3	9.3	8.5	8.4	8.3	9.2	10.3	8.3	9.2	8.9	9.3	8.9
**Urea**	6.9	6.7	6.5	5.8	6.1	6.0	6.8	6.5	6.7	6.7	6.8	7.1	6.6	6.3	6.8	6.5	6.7	6.5	7.3	6.7	6.6
**Uridine**	5.0	4.8	5.2	4.9	4.8	5.3	4.9	4.8	5.2	5.2	5.3	5.2	4.9	5.1	4.6	4.8	4.9	5.1	4.9	5.0	5.1
**Uridine-5-diphosphate**	2.3	1.9	2.7	2.0	2.4	2.4	2.2	2.2	2.6	2.2	2.4	2.6	1.6	2.2	1.8	2.4	2.3	2.0	2.6	1.6	2.0

Table S2. Metabolite features from LC-MS included in random forest modelling. Data are median and log transformed and listed under SiteID.

	SiteID																						
Metabolite	25	301	302	306	312	314	326	395	465	698	848	931	933	936	937	940	945	947	954	960	963	985	990
Ammonia	10.4	10.2	9.9	9.8	10.6	10.2	10	9.8	10.5	10.2	10.6	10.4	10.3	9.5	10.6	10.1	10.8	9.5	10	10.2	10.2	10.1	10.1
Histidine	7.8	7.7	7.4	7.4	8	7.7	7.3	7.4	8	7.5	8	7.9	7.6	6.9	7.9	7.1	8.1	7.1	7.5	7.5	7.8	7.4	7.5
Asparagine	9.8	9.9	9.7	9.9	10.5	9.7	10	9.8	10.1	9.9	10.1	9.9	10.1	9.5	10.2	9.8	10.4	9.9	10	10.2	10.1	9.7	10
Norepinephrine	1.2	3.4	1.6	1.2	1.7	1.1	1.1	1.5	1.3	0.9	1.5	1.2	1	1.3	1.1	1.3	1.6	1.6	1	0.9	1.2	1	1.1
Taurine	6.9	6.9	7.4	7.2	7.8	7.1	6.9	7.4	7.4	6.9	7.5	7.4	6.9	7.2	6.9	7.3	7.6	6.6	6.8	6.8	7.2	6.9	7
Arginine	8.6	8.4	8.2	8.3	8.9	8.7	8.2	8.2	8.8	8.5	8.8	8.7	8.7	8	8.7	8.3	8.8	8.4	8.1	8.7	8.5	8.5	8.4
Serine	9.9	9.9	9.6	9.7	10.3	9.8	9.9	9.6	10.1	9.7	10.1	10	10.1	9.4	10.4	9.5	10.2	10	9.9	9.9	9.8	9.9	9.8
Ethanolamine	8.3	8.2	7.7	7.7	8.4	7.8	8	7.8	8.2	7.8	8.1	8.5	8.1	7.3	8.6	7.6	8.5	7.8	8.2	7.8	8.2	7.8	8
Cystamine	6.4	6.2	6	6.1	6.7	6.3	6.2	6.1	6.5	6.2	6.6	6.5	6.6	5.7	6.7	5.9	6.7	6.4	6.3	6.4	6.3	6.1	6.2
Glycine/Amino 2-propanol	10.7	10.5	10.3	10.4	11	10.6	10.4	10.4	10.8	10.5	10.9	10.8	10.9	9.9	11	10.1	10.9	10.7	10.6	10.7	10.6	10.4	10.5
Cystine	1.7	2.5	2	2.2	2.9	2.1	1.7	3.2	1.8	2.1	2.5	2.2	2.3	3.4	2	3.1	2.9	2.3	2	3.2	1.5	2.8	2.4
Homoserine/Threonine	5.1	6.1	5.5	5.8	6.4	5.9	5.5	6.9	5.5	5.7	6.2	5.9	5.7	7	5.8	6.8	6.7	6	5.7	6.9	5.1	6.5	5.9
Pyridoxamine	3.2	3	2.6	2.5	3.1	3.1	2.9	2.1	3.2	2.9	3.7	3.2	3.2	2	3.4	2.6	3.3	2.7	2.9	3	2.7	2.5	3.1
Aspartate	8.6	9.1	8.2	9	9.1	8.6	8.2	8.2	8.7	8	9	8.8	9	8.2	9.3	8.1	8.4	8.7	9	8.4	9.2	8.9	8.7
Agmatine	4	4	6.4	6.2	6.3	4.6	4.2	4.5	3.8	4.3	4.1	3.7	4.6	3.6	4.5	3.9	5.3	7.2	4.4	6.6	3.8	5.9	3.5
Citrulline	5	6.1	4.4	4.7	5.1	4.9	4	4.7	5.1	4.5	5.2	5	5.1	4.8	5	5.5	5.1	5.1	4.9	5.2	5.3	5	5.8
Glutamate	10.6	10.6	10.3	10.4	11.1	10.3	10.4	10.2	10.5	10.1	10.4	10.7	10.6	9.5	10.8	9.9	10.7	10.6	10.4	10.2	10.8	10.1	10.2
Alanine	10.6	10.3	9.7	9.4	10.4	9.9	10	9.6	10.6	9.9	10.5	10.2	10.2	9.5	10.5	10	10.9	10	9.9	10	10	10	10.1
Ethylamine	5.9	5.8	5.5	5.2	6.2	5.6	5.8	5.5	6.2	5.6	6.1	5.9	5.7	5.4	6.3	5.8	6.8	5.1	5.6	5.8	5.8	5.6	5.8
2-Aminobutyric acid/GABA	7.3	8	7	6.9	6.9	7.3	7	8.2	6.9	7.1	6.9	6.9	7.1	7.1	7.3	7.1	7.5	6.9	6.9	7	6.9	7	7
Methionine	4	4	3.7	3.4	4.3	3.9	3.9	3.7	4.3	3.9	4.3	4.1	3.9	3.5	4.4	4	4.9	3.6	3.7	3.9	4	3.8	3.9
Proline	10.5	10	10.1	10.2	10.9	10.1	10.2	9.8	10.6	10.2	10.3	10.5	10.3	9.6	10.5	9.6	10.7	10.4	10.1	9.8	10.3	9.9	9.9

	SiteID																						
Metabolite	25	301	302	306	312	314	326	395	465	698	848	931	933	936	937	940	945	947	954	960	963	985	990
Homocysteine	3.6	2.7	0.3	3.1	2.9	3	2.2	2	2.6	2.2	3.2	2.5	2.5	3.4	2.7	1.9	3.6	3	4.6	3.2	3.5	4.2	3
Ornithine	8.1	8.1	7.6	8.1	7.8	7.8	8.3	7.7	8.2	7.7	8.3	8.5	8.3	7.4	8.3	7.6	7.9	7.7	8.2	8.2	8	7.8	8.1
Dihydroxyphenylalanine	4.2	3.6	4.1	3.8	4.4	4.2	3.8	3.8	4.1	3.8	4.3	4.3	3.8	3.4	4.5	3.8	4.9	4.6	3.5	3.8	4.1	3.8	4.5
Trypanothione	2.3	2.7	2.4	2.2	2.9	2.5	1.9	2.3	2.8	2.5	2.7	2.6	1.8	2.2	2.9	2.6	3.5	1.9	2.3	2.3	2.2	2.5	2.4
Lysine	7	7.1	6.9	7	7.2	6.9	7	6.9	7	6.9	7	7.1	7	6.7	7.3	6.9	6.9	7	7	7.1	6.7	7.1	6.9
Putrescine	5.6	5.3	5.3	5.8	6.2	5.9	5.2	5.7	5.7	4.9	5.5	6.1	6	5	5.5	4.9	5.1	6.4	5.9	6.1	5.4	5.7	5.7
Cysteamine	1.9	1.8	1.2	1.4	1.7	1.7	1.5	1.4	1.7	1.7	1.8	1.8	1.9	1.3	1.9	1.5	2	1.3	1.7	1.6	1.4	1.6	1.6
5-Hydroxytryptophan	5	5.8	5	5.5	5.8	6	5.1	5.1	5.9	5.5	6.3	5.6	5.5	5.2	5.7	5.4	6.5	4.9	5.3	6.1	5.5	5.3	5.1
3-Hydroxytyramine/Octopamine	5.8	6.4	5.9	6	6.9	5.5	5.8	6.3	6.2	5.2	5.8	6.7	5.7	5.5	4.7	5.6	6.2	6	5.7	5.8	7	5.7	6.3
Tyrosine	10.6	10.5	10.5	10.2	11.2	10.2	10.4	10.3	10.6	10.3	10.6	11.1	10.3	9.4	10.6	10	11	10.6	10.4	10.1	11.3	10.2	10.3
Indole 3-butyric acid	8.7	8.6	7.9	7.7	8.8	8.1	8.3	8.1	9	8.2	8.7	8.5	8.5	7.6	8.8	8.3	9.3	8.1	8.2	8.4	8.3	8.2	8.4
Valine	11.1	10.9	10.8	10.7	11.4	10.9	10.9	10.7	11.2	10.9	11.2	11.3	11.1	10.2	11.3	10.4	11.4	10.9	10.9	10.9	11.1	10.9	10.8
Cadaverine	8.3	8.4	7.9	8.5	7.9	8.8	7.4	7.4	8	8.3	8.5	8.5	8.6	7.9	8.8	8.2	8.2	7.3	8	8.5	7.8	7.9	8.1
Serotonin	2.5	2.9	1.1	2.4	4.4	2.4	2.5	2.1	3.3	2.2	2.3	2.6	2.2	2.6	3.2	4.1	1.4	2.6	2.5	2.4	2.2	4.1	2.7
Spermidine	0.9	1.4	0.8	1.5	1.3	1.2	1.2	0.6	1.1	0.9	0.8	1.6	1.3	0.7	0.7	0.7	0.8	1.2	1	1.4	1.7	0.8	1.4
Tyramine/PABA	11	10.8	10.6	10.3	11.2	10.7	10.9	10.5	11.2	10.8	11.2	11	10.9	10.4	11.3	10.9	11.9	10.7	10.7	10.8	10.8	10.8	10.8
3-Methoxytyramine	5.9	5.7	5.3	5.2	5.8	5.3	5.7	5.2	5.9	5.3	5.8	5.8	5.8	4.9	6.1	5.3	6	5.5	5.5	5.4	5.6	5.5	5.5
4-Hydroxyproline	11.1	11.6	10.4	10.6	11.1	11.5	10.5	10.5	10.9	10.6	10.9	10.9	11.3	10.3	11.1	10.3	11	10.7	10.8	10.6	10.8	11	10.5
Isoleucine	11.7	11.5	11.4	11.3	12	11.4	11.4	11.4	11.6	11.3	11.6	11.9	11.7	11	11.9	11	11.8	11.5	11.3	11.5	11.7	11.5	11.4
Leucine	10.9	11.3	10.4	10.7	11	10.7	10.9	10.5	10.9	10.7	11	11	11	10.3	11	10.4	11	10.7	10.8	10.6	10.8	11	10.6
Kynurenine/Lanthionine	4.2	4.4	3.9	4	4	4.1	3.8	4.3	4.2	3.4	4.2	4.5	4.6	3.6	4.5	3.6	3.8	4.1	4.3	4.1	3.8	3.4	4.1
Serotonin	5.3	5	4.8	4.6	5.4	5	5.1	4.7	5.4	5.1	5.4	5.3	5.2	4.6	5.5	5.1	6	4.9	4.9	5	5	5	5
Phenylalanine	10	10	9.8	9.7	10.2	9.6	10	9.6	9.9	9.5	9.9	10.2	10.1	9.4	10.4	9.4	10	9.9	9.9	9.7	9.8	9.9	9.7

	SiteID																						
Metabolite	25	301	302	306	312	314	326	395	465	698	848	931	933	936	937	940	945	947	954	960	963	985	990
Tryptophan	8.6	8.5	8.1	8	8.6	8	8.2	8	8.5	8	8.2	8.7	8.5	7.7	8.7	7.8	8.5	8.2	8.3	8.1	8.7	8.3	8.3
Cystathionine	6	5.4	4	4.5	5.9	5.3	6	5.4	6.4	5.3	6	5.8	6.2	4.4	6.3	5.4	6.3	5.1	5.4	5.9	6.1	4.7	5.5
Cysteine/Phenyethylamine	5.6	3.2	4.3	5	4.2	3.2	2.2	3.8	4.2	2.9	5.5	3.2	4.5	2.4	5.7	3.1	3	6.5	7.1	4.4	2.9	3.6	3.2
Tryptamine	1.3	1.4	2.5	1.9	1.7	2.7	1.5	2	2.1	1.4	2	1.6	1.7	1.4	2.3	1.5	2	1.6	2.2	1.3	1.8	1.6	1.5

Table S3. Location of 21 wetlands where *Procladius villosimanus* were sampled for metabolomic extractions. Including the number of replicates (pooled individuals) for each site.

SiteID	Latitude	Longitude	Site description	Melways Ref	Replicate extractions
25	-37.77616897	145.3599688	Platypus Wetlands at Hull Rd, Lilydale	38 G10	2 (9, 11)
301	-37.714001	145.055827	Gresswell Reserve at Moonstone Walk, Bundoora	19 J5	2 (6, 6)
302	-37.8814907	144.7381911	Broadwalk Wetland at Central Park Ave, Point Cook	207 G2	1 (6)
306	-37.706695	145.047838	Mt Cooper Wetland, Bundoora	19 G3	3 (10, 9, 10)
312	-37.69249	144.93688	Jack Roper Reserve Lake at Camp Rd, Broadmeadows	7 B10	3 (10, 9, 9)
314	-37.76116144	144.9238398	Queens Park Lake at the Strand, Moonee Ponds	28 J6	2 (15, 15)
326	-37.82372703	145.3002934	Bungalook Creek RB at Canterbury Rd, Bayswater North	51 C11	1 (6)
395	-37.637193	144.947852	Austrak RB at Regional Drv, Somerton	180 D8	1 (8)
465	-37.761827	144.77923	Station Waters Wetland at Waterview Drive, Cairnlea	25 F6	3 (10, 11, 8)
698	-37.888398	144.620568	Amber Place Wetland, Wyndham Vale	204 K4	3 (10, 10, 10)
848	-37.680679	145.053568	Botanica Blvd opp Pride Ave (North Pond), Bundoora	9 H7	3 (9, 10, 10)
931	-37.9910215	145.1534952	Cheltenham Rd Rb, U/S Chelt. Rd, Dandenong South	89 A9	3 (8, 8, 9)
936	-38.007559	145.09918	Epsom Estate, Hutchins Close, Mordialloc	92 H1	2 (9, 9)
937	-37.901932	145.30779	Gilmour Park Rb, Ferndale Rd, Upper Ferntree Gulley	74 E8	3 (9, 10, 11)
940	-37.91454825	144.7771795	Saltwater Coast Wetlands, Point Cook	208 F11	3 (8, 8, 8)
945	-37.73692361	144.7624586	Kinterbury Drive Wetland, Kings Park	13 C11	1 (5)
954	-37.864257	144.809432	Mt St Joseph Wetlands, Civic Pde, Altona	54 C10	2 (14, 14)
960	-37.61973613	144.9314303	Paroo Ave, Roxborough Park (RB5)	179 K3	3 (10, 9, 9)
963	-37.64974542	144.947055	National Business Park RB at Link Drive, Campbellfield	180 D11	3 (10, 10, 10)
985	-37.91003601	145.2618964	Lysterfield West Rb, Lysterfield	73 E10	2 (8, 8)
990	-37.85012901	145.2909511	Old Joes Creek Rb, Bayswater North	50 K10	3 (10, 10, 9)

Table S4. Quality assurance and control for organics analysis by GC-MS in wetland sediments, including mean spike Recovery and LORs. This analysis was performed by University of Melbourne Chemistry Department.

Compound	Mean spike recovery (%)	LOR (ppb)
3,4-dichloro-	51	<2
3,4-dichlorobenzenamine	175	<20
Atrazine	76	<5
Atrazine deisopropyl	23	<50
Atrazine desethyl (DEA)	33	<20
Azoxystrobin	108	<75
Bifenthrin	62	<2
Boscalid	85	<10
Carbaryl[1]	431	<5
Chlorpyrifos	95	<10
Cypermethrin 1/4	137	<200
Cypermethrin 2/4	64	<200
Cypermethrin 3/4	59	<200
Diethyltoluamide (DEET)	64	<5
Difenoconazole 1/2	73	<75
Difenoconazole 2/2	74	<75
Dimethoate	27	<50
Dimethomorph 1/2	100	<75
Dimethomorph 2/2	74	<100
Diuron as 3,4-DIB+3,4-	403	<2.5
Fenamiphos	114	<10
Fipronil	146	<5
Indoxacarb	224	<50
Iprodione1	155	<25
Kresoxim-methyl	101	<2
Linuron	212	<50
Malathion[1]	173	<5
Metalaxyl	92	<5
Metolachlor	96	<2
Myclobutanil	105	<15
Permethrin 1/2	63	<10
Permethrin 2/2	46	<20
Prochloraz	79	<200
Prometryn	95	<2
Pyraclostrobin	165	<40
Pyrimethanil	74	<1
Simazine	60	<5
Triclosan	143	<10
Trifloxystrobin	107	<5

[1] these compounds breakdown on GC with the proportional breakdown in samples differentially from pure standards.

Table S5. Quality assurance and control for metal and hydrocarbon analysis in wetland sediments. Method limits of reporting (LOR), replicate analytical analysis (expressed as relative standard deviation (RPD), %, n = 43) (mg/kg) and analytical precision (range of spike recovery, %, n = 23). Analysis was performed by Australian Laboratory Services.

Analyte	Method Limit of reporting (mg/kg)	Laboratory QA/QC	
		Duplicate (RSD%)	Spike recovery (%)
Arsenic	<5	0-10	79 - 113
Cadmium	<1	0-10	87 - 115
Chromium	<2	0-8.3	89 - 113
Copper	<5	0-11.3	90 - 116
Lead	<5	0-10.9	85 - 107
Mercury	<0.1	0-36.5	85 - 103
Nickel	<2	0-8.6	89 - 111
Silver	<2	0-4.6	80 - 108
TPH	<50	0-23.1	65 - 131
Zinc	<5	0-10.4	89 - 111

Table S6. Annotation of metabolite features detected through PyMS analysis of GC-MS data, including database top hits, retention time, quant and second ions of metabolic features. Grey text indicates peaks that were removed because they aligned with authentic standard identified peaks (indicated in Table S1).

Metabolite name	Quant ion	Second ion	Retention time (sec)	PBM top hit	PBM %match	NIST top hit	NIST %match
1,2,5-Oxadiazole-3,4-dicarboxamide	217	186	747.95	1,2,5-Oxadiazole-3,4-dicarboxamide, 4TMS derivative	83	not identified	
17-beta-Acetoxyandrostane	243	169	681.94	Androstan-17-ol, acetate, (17.beta.)- (CAS) $$ 17.BETA.-ACETOXY ANDROSTANE $$ 17.beta.-Acetoxyandrostane $$ Androstan-17.beta.-ol, acetate	97	Inositol, 1,2,3,4,5,6-hexakis-O-(trimethylsilyl)-, scyllo-	63.6
1H-Cycloprop[e]azulen-4-ol	361	204	856.2	1H-Cycloprop[e]azulen-4-ol, decahydro-1,1,4,7-tetramethyl-, [1aR-(1a.alpha.,4.alpha.,4a.beta.,7.alpha.,7a.beta.,7b.alpha.)]-(CAS) $$ Ledol $$ d-Ledol $$ (+)-Ledol $$ 1H-Cycloprop[e]azulen-4-ol, decahydro-1,1,4,7-tetramethyl-, (1aR,4R,4aS,7R,7aS,7bS)- $$	92	not identified	
2-Anthracenecarboxylic acid,	310	204	850.99	Methyl 3-methoxy-1-methylanthraquinone-2-carboxylate $$ 2-Anthracenecarboxylic acid, 9,10-dihydro-3-methoxy-1-methyl-9,10-dioxo-, methyl ester (CAS)	72	not identified	
2-Methylcortisol	103	75	788.45	2-Methylcortisol	90	not identified	
2-Piperidone	128	115	533.68	2-Piperidone, 1-(trimethylsilyl)-3-[(trimethylsilyl)amino]-(CAS) $$ N,N'-DI-TMS-2,5-DIAMINOVALEROLACTAM	93	2-Piperidone, 1-(trimethylsilyl)-3-[(trimethylsilyl)amino]-	84.2
2,6-di-t-butyl-4-(bromomethyl)phenol	103	75	814.53	2,6-DI-T-BUTYL-4-(BROMOMETHYL)PHENOL	90	not identified	
3-Bromo-N-(3,5-dichlorophenyl)benzamide	103	75	791.73	3-Bromo-N-(3,5-dichlorophenyl)benzamide, TMS derivative	98	not identified	
3-phosphoglyceric acid	299	227	627.58	[439183] 3-phosphoglyceric acid [16.504]	91	3,5-Dioxa-4-phospha-2-silaoctan-8-oic acid, 2,2-dimethyl-4,7-	49

**Metabolite name**	**Quant ion**	**Second ion**	**Retention time (sec)**	**PBM top hit**	**PBM %match**	**NIST top hit**	**NIST %match**
						bis[(trimethylsilyl)oxy] -, trimethylsilyl	
3,4-dihydroxyphenylacetic acid	179	75	636.6	[547] 3,4-dihydroxyphenylacetic acid_ [16.728]	91	Benzeneacetic acid, 3,4-bis[(trimethylsilyl)oxy] -, trimethylsilyl ester	98.4
3,4-dihydroxyphenylacetic acid	217	103	637.88	3,4-Dihydroxyphenylacetic Acid, 3TMS derivative	87	not identified	
5-Hydroxytryptophol	290	192	723.21	5-Hydroxytryptophol, 3TMS derivative	72	trimethylsilyl 1-trimethylsilyl-5-trimethylsiloxy-3-(2-trimethylsilylamino)indolepropionate	85.5
5.alpha.-androstane	103	75	801.3	Silane, [[(5.alpha.,11.beta.)-androsta-2,16-diene-11,17-diyl]bis(oxy)]bis[trimethyl- (CAS) $$ 5.ALPHA.-ANDROSTANE-2,17-DIENE-11.BETA.,17-DIOL TMS $$ 11.BETA.,17-DIHYDROXY-5.ALPHA.-ANDROSTANE-2,17-DIENE 11,17-TMS	90	not identified	
5.alpha.-androstane	117	103	752.53	Silane, [[(5.alpha.,11.beta.)-androsta-2,16-diene-11,17-diyl]bis(oxy)]bis[trimethyl- (CAS) $$ 5.ALPHA.-ANDROSTANE-2,17-DIENE-11.BETA.,17-DIOL TMS $$ 11.BETA.,17-DIHYDROXY-5.ALPHA.-ANDROSTANE-2,17-DIENE 11,17-TMS	90	not identified	
5.alpha.-androstane	221	207	758.33	Silane, [[(5.alpha.,11.beta.)-androsta-2,16-diene-11,17-diyl]bis(oxy)]bis[trimethyl- (CAS) $$ 5.ALPHA.-ANDROSTANE-2,17-DIENE-11.BETA.,17-DIOL TMS $$ 11.BETA.,17-DIHYDROXY-5.ALPHA.-ANDROSTANE-2,17-DIENE 11,17-TMS	90	not identified	

Metabolite name	Quant ion	Second ion	Retention time (sec)	PBM top hit	PBM %match	NIST top hit	NIST %match
9H-Purine	353	294	684.7	9H-Purine, 9-(trimethylsilyl)-2,6-bis[(trimethylsilyl)oxy]- (CAS) $$ XANTHINE-TRI-TMS $$ XANTHINE, O2,O6,9-TRIS(TRIMETHYLSILYL) $$ XANTHINE-N2,O6,9-TRITMS	93	7-(Trimethylsilyl)-2,6-bis[(trimethylsilyl)oxy] -7H-purine	85.5
adenosine-5-monophosphate	315	169	877.34	[224] adenosine-5-monophosphate 1 [26.837]	74	not identified	
alpha-D-Galactopyranoside	103	74	852.49	.alpha.-D-Galactopyranoside, methyl 2,3-bis-O-(trimethylsilyl)-, cyclic butylboronate (CAS) $$ A-GALACTOPYRANOSIDE-1-METHYL-4,6-BUTYLBORONATE-2,3-DITMS	98	not identified	
Androstan-17-ol, acetate	243	204	764.6	Androstan-17-ol, acetate, (17.beta.)- (CAS) $$ 17.BETA.-ACETOXY ANDROSTANE $$ 17.beta.-Acetoxyandrostane $$ Androstan-17.beta.-ol, acetate	92	not identified	
Benzenebutanoic acid	208	204	757.29	Benzenebutanoic acid, .gamma.-oxo-.alpha.,2-bis[(trimethylsilyl)amino]-3-[(trimethylsilyl)oxy]- (CAS) $$ 3 HYDROXY KYNURENINE TRITMS	91	Benzenebutanoic acid, _-oxo-_-amino-, 2-(trimethylsilyl)amino-, 3-(trimethylsilyl)oxy-, trimethylsilyl ester	85
beta-Alanine	102	75	452.6	.beta.-Alanine, N-(trimethylsilyl)-, trimethylsilyl ester (CAS) $$ N,O-DI-TMS-.BETA.-ALANINE $$ TRIMETHYLSILYL ESTER OF N-TRIMETHYLSILYL-.BETA.-ALANINE	60	Glycine, N-acetyl-N-(trimethylsilyl)-, trimethylsilyl ester	70
beta-Alanine	102	86	447.1	.beta.-Alanine, N-(trimethylsilyl)-, trimethylsilyl ester (CAS) $$ N,O-DI-TMS-.BETA.-ALANINE $$ TRIMETHYLSILYL ESTER OF N-TRIMETHYLSILYL-.BETA.-ALANINE	95	_-Alanine, N-(trimethylsilyl)-, trimethylsilyl ester	67.8
beta-Alanine	174	160	522.98	beta-Alanine	38	beta-Alanine	76.5
Citric acid	273	75	630.99	[311] citric acid [16.615]	91	1,2,3-Propanetricarboxylic acid, 2-	96.5

Metabolite name	Quant ion	Second ion	Retention time (sec)	PBM top hit	PBM %match	NIST top hit	NIST %match
						[(trimethylsilyl)oxy]-, tris(trimethylsilyl) ester Formula: C18H40O7Si4	
Citrulline 2	157	142	634.33	[9750] citrulline 2 [16.691]	86	Citrulline, tetra(trimethylsilyl)-	31
Cortisone	204	129	779.58	Cortisone	91	not identified	
D-(-)-3-Phosphoglyceric acid	357	299	615.42	D-(-)-3-Phosphoglyceric acid	91		
D-(-)-Erythrose	201	102	652.18	D-(-)-Erythrose, tris(trimethylsilyl) ether, methyloxime (anti)	84	not identified	
D-Maltose	204	103	811.94	D-Maltose Pk1	72		
D-Maltose	204	117	824.76	D-Maltose Pk1	91		
D-Maltose	361	204	830.32	D-Maltose Pk2	91		
D-Maltose	361	217	817.4	D-Maltose Pk2	78		
D-Talose	319	205	656.45	D-Talose Pk2	91		
D-Talose	319	205	649.69	D-Talose Pk1	91		
DL-Isoleucine	86	75	445.31	[791] DL-isoleucine 1 [8.576]	90	I-Isoleucine, trimethylsilyl ester	77.9
exclude	117	100	623.4	not identified		not identified	
exclude	141	69	505.91	not identified		not identified	
exclude	230	114	534.4	not identified		not identified	
exclude	234	128	592.29	not identified		not identified	
exclude	242	70	606.88	3-thio-1,2,5,6,11,11b-hexahydro-3H-indo[2,3-a]indolizine $$ 3H-Indolizino[8,7-b]indole-3-thione, 1,2,5,6,11,11b-hexahydro-, (.+-.)-	74	6-Azauracil, N,N'-bis(trimethylsilyl)-	31.2
exclude	292	205	553.77	not identified		not identified	

**Metabolite name**	**Quant ion**	**Second ion**	**Retention time (sec)**	**PBM top hit**	**PBM %match**	**NIST top hit**	**NIST %match**
exclude	305	260	604.34	not identified		not identified	
exclude	71	58	535.44	not identified		not identified	
exclude	75	74	379.62	not identified		not identified	
exclude	76	50	422.9	not identified		not identified	
exclude	84	70	653.55	not identified		not identified	
Fumaric acid	245	75	497.67	Fumaric acid	91		
Fumaric acid	298	132	496.76	[444972] fumaric acid [10.94]	90	2-Butenedioic acid (E)-, bis(trimethylsilyl) ester	94.9
Glucopyranose	204	191	667.61	Glucopyranose, 5TMS derivative	95	not identified	
Glucose-6-phosphate	387	299	749.88	Glucose-6-phosphate Pk1	72		
Glucose-6-phosphate	387	299	746.96	Glucose-6-phosphate Pk2	72		
Glyceric acid	189	103	488.87	[439194] glyceric acid [10.735]	91	Propanoic acid, 2,3-bis[(trimethylsilyl)oxy]-, trimethylsilyl	80.8
Glycerol	205	117	470.25	[753] glycerol [9.941]	91	Glycerol, tris(trimethylsilyl) ether	82.4
Glycerol 1-phosphate	70	56	616.3	[754] glycerol 1-phosphate [16.056]	90	Phosphoric acid, bis(trimethylsilyl) 2,3-bis[(trimethylsilyl)oxy] propyl ester	90.8
Glycine	102	66	424.65	Glycine Pk1	87		
Glycine	174	86	485.16	Glycine Pk2	91		
Inosine	217	103	798.3	[6021] inosine [23.396]	91	Inosine, tetrakis(trimethylsilyl) ether	97.8

Metabolite name	Quant ion	Second ion	Retention time (sec)	PBM top hit	PBM %match	NIST top hit	NIST %match
Isopropyl beta-D-1-thiogalactopyranoside	217	204	669.12	[656894] isopropyl beta-D-1-thiogalactopyranoside_ [19.097]	83	not identified	
L-Alanine	116	59	415.35	L-Alanine	86		
L-Alanine	116	75	419.3	L-Alanine	90		
L-Asparagine	115	99	593.9	[236] L-asparagine 2 [14.984]	91	L-Asparagine, N,N2-bis(trimethylsilyl)-, trimethylsilyl ester	92.7
L-Asparagine	116	75	593.87	L-Asparagine	91		
L-Aspartic acid	207	99	548.65	L-Aspartic acid, 3TMS derivative	83	L-Aspartic acid, N-(trimethylsilyl)-, bis(trimethylsilyl) ester	70.4
L-Aspartic acid	232	100	549.49	L-Aspartic acid	72		
L-glutamic acid	246	128	578.64	[33032] L-glutamic acid 2 [14.398]	91	Glutamic acid, N-(trimethylsilyl)-, bis(trimethylsilyl) ester, L-	92.2
L-Glutamine 3	156	155	622.16	[738] L-glutamine 3 [16.092]	91	l-Glutamine, tris(trimethylsilyl) deriv.	97.3
L-Isoleucine	158	100	479.63	L-Isoleucine	90		
L-Isoleucine	89	79	444.5	L-Isoleucine (CAS) $$ Isoleucine, L- $$ DL-ISO-LEUCINE $$ ISOLEUCINE, D,L- $$ L(+)-Isoleucine $$ Isoleucine $$ Norvaline, 3-methyl- $$ erythro-L-Isoleucine $$ 2-Amino-3-methylvaleric acid $$ Norvaline, 3-methyl-, erythro- $$ 2-Amino-3-methylpentanoic acid	90	l-Isoleucine, trimethylsilyl ester	71.9
L-Lysine	174	156	659.12	L-Lysine Pk2	91		

Metabolite name	Quant ion	Second ion	Retention time (sec)	PBM top hit	PBM %match	NIST top hit	NIST %match
L-Lysine	84	74	602.65	L-Lysine, N2,N6-bis(trimethylsilyl)-, trimethylsilyl ester (CAS) $$ N,N',O-TRITMS LYSINE $$ Lysine, N2,N6-bis(trimethylsilyl)-, trimethylsilyl ester, L-	95	l-Lysine, N2,N6-bis(trimethylsilyl)-, trimethylsilyl ester	95.4
L-Methionine	176	128	552.36	L-Methionine	80		
L-Ornithine	142	128	632.37	L-Ornithine	91		
L-Ornithine	142	70	577.58	[6262] L-ornithine 1 [14.349]	91	Ornithine, tri-TMS	96.5
L-Ornithine	188	100	577.4	[6262] L-ornithine 1 [14.349]	87	Ornithine, tri-TMS	97.4
L-Proline	142	75	483.6	L-Proline Pk1	80		
L-Proline	370	102	482.76	[145742] L-proline 2 [10.321]	86	L-Proline, 1-(trimethylsilyl)-, trimethylsilyl ester	94.7
L-Serine	204	100	499.3	L-Serine Pk2	91		
L-Serine	340	79	499.71	[5951] L-serine 2 [11.174]	74	Serine, N,O-bis(trimethylsilyl)-, trimethylsilyl	75.9
L-Threonine	117	75	480.9	L-Threonine Pk1	91		
L-Threonine	218	117	507.36	L-Threonine Pk2	91		
L-Tryptophan	202	74	730.74	L-Tryptophan Pk2	91		
L-Tyrosine	218	100	664.68	L-Tyrosine Pk2	91		
L-Tyrosine	218	171	678.5	L-Tyrosine Pk2	91		
L-Tyrosine	99	71	663.97	[6057] tyrosine 2 [17.871]	90	L-Tyrosine, N,O-bis(trimethylsilyl)-, trimethylsilyl ester	92.1
L-Valine	144	100	454.56	L-Valine	91		
Lactic Acid	117	66	392.91	Lactic Acid	72		
Lactic Acid	117	66	401.87	Lactic Acid	91		
Lactic Acid	117	75	399.62	Lactic Acid	91		

Metabolite name	Quant ion	Second ion	Retention time (sec)	PBM top hit	PBM %match	NIST top hit	NIST %match
Lactic Acid	117	75	405.77	Lactic Acid	91		
Lactic Acid	117	88	406.95	Lactic Acid	91		
Lactic Acid	174	74	403.53	[107689] L-(+) lactic acid [6.851]	91	not identified	
Maltose	204	129	792.9	[6255] maltose 2 [24.915]	86	not identified	
Maltotriose	204	103	1039.4	[192826] maltotriose 2 [30.668]	87	not identified	
Maltotriose	204	103	980.27	Maltotriose Pk1	80		
Maltotriose	204	103	977.6	Maltotriose Pk1	91		
Mannitol	319	205	656.45	Mannitol	91		
methyl-beta-D-Galactopyranoside	204	133	646.99	[94214] methyl-beta-D-galactopyranoside__ [16.935]	79	not identified	
Myo-Inositol	217	191	698.19	Myo-Inositol, 6TMS derivative	95	Myo-Inositol, 1,2,3,4,5,6-hexakis-O-(trimethylsilyl)-	74.4
N-acetyl-D-mannosamine	205	202	695.58	[899] N-acetyl-D-mannosamine 2 [19.339]	72	N-Acetyl-D-glucosamine, tetrakis(trimethylsilyl) ether, methyloxime (syn)	64.4
N-acetyl-L-histidine	224	182	708.95	[273260] N-acetyl-L-histidine 2 [19.507]	83	not identified	
noise	100	74	517.44	noise		noise	
noise	100	75	544.21	noise		noise	
noise	100	75	611.9	noise		noise	
noise	100	89	464.84	noise		noise	
noise	100	99	581.65	noise		noise	
noise	102	84	582.4	noise		noise	
noise	103	102	722.46	noise		noise	
noise	103	75	778.24	not identified		not identified	

**Metabolite name**	**Quant ion**	**Second ion**	**Retention time (sec)**	**PBM top hit**	**PBM %match**	**NIST top hit**	**NIST %match**
noise	103	75	786.51	1,2,3,4,6,7,8,8a-Octahydronaphthalene-6,7-diol, 5,8a-dimethyl-3-isopropenyl-, cyclic carbonate, trans-	91	not identified	
noise	107	106	382.42	noise		noise	
noise	114	79	463.31	noise		noise	
noise	115	100	566.82	not identified		not identified	
noise	117	101	461.73	noise		noise	
noise	117	79	385.32	noise		noise	
noise	117	79	381.34	noise		noise	
noise	121	79	394.87	noise		noise	
noise	125	79	432.66	noise		noise	
noise	128	58	545.9	noise		noise	
noise	128	99	609.97	noise		noise	
noise	130	75	440.28	noise		noise	
noise	130	75	429.61	not identified		not identified	
noise	131	79	550.86	noise		noise	
noise	142	75	571.27	noise		noise	
noise	148	55	574.74	noise		noise	
noise	148	59	439.27	noise		noise	
noise	148	66	426.36	noise		noise	
noise	154	74	517.88	noise		noise	
noise	156	100	460.71	noise		noise	
noise	156	55	431.79	noise		noise	
noise	167	156	694.1	noise		noise	
noise	170	79	457.24	noise		noise	
noise	171	100	546.9	noise		noise	
noise	171	99	596.51	noise		noise	

Metabolite name	Quant ion	Second ion	Retention time (sec)	PBM top hit	PBM %match	NIST top hit	NIST %match
noise	173	156	605.92	not identified		not identified	
noise	174	100	589.2	noise		noise	
noise	174	102	580.9	noise		noise	
noise	174	82	583.43	noise		noise	
noise	179	75	547.98	noise		noise	
noise	183	99	532.36	noise		noise	
noise	185	103	569.6	noise		noise	
noise	204	171	680.31	noise		noise	
noise	207	191	389.14	noise		noise	
noise	217	103	570.2	noise		noise	
noise	217	115	573.37	noise		noise	
noise	217	202	692.69	noise		noise	
noise	221	117	504.38	noise		noise	
noise	221	204	784.21	not identified		not identified	
noise	221	205	595.8	noise		noise	
noise	223	117	685.42	noise		noise	
noise	239	75	514.59	Pentanedioic acid, bis(trimethylsilyl) ester (CAS) $$ GLUTARIC ACID-DITMS $$ BISTRIMETHYLSILYL GLUTARIC ACID $$ GLUTARICACID-DITMS $$ pentan-di-oic acid bis(trimethylsilyl) ester $$ Bis(trimethylsilyl) glutarate	47	noise	
noise	243	79	530.32	noise		noise	
noise	246	70	560.33	noise		noise	
noise	247	75	526.47	noise		noise	
noise	255	75	502.65	noise		noise	
noise	280	79	681.25	noise		noise	

Metabolite name	Quant ion	Second ion	Retention time (sec)	PBM top hit	PBM %match	NIST top hit	NIST %match
noise	293	217	678.98	noise		noise	
noise	298	207	508.55	noise		noise	
noise	299	133	473.9	noise		noise	
noise	318	75	590.32	noise		noise	
noise	319	128	718.36	noise		noise	
noise	319	129	572.57	noise		noise	
noise	350	148	529.29	5-(1',3'-Dithian-2'-yl)-11-methyl-6H-pyrido[4,3-b]carbazole $$ 6H-Pyrido[4,3-b]carbazole, 5-(1,3-dithian-2-yl)-11-methyl-	72	noise	
noise	380	204	711.5	noise		noise	
noise	66	59	410.7	noise		noise	
noise	69	52	521.55	noise		noise	
noise	69	55	519.29	noise		noise	
noise	74	50	520.77	noise		noise	
noise	75	66	408.5	noise		noise	
noise	75	70	421.77	noise		noise	
noise	75	74	411.73	noise		noise	
noise	75	74	396.2	noise		noise	
noise	75	74	460.6	noise		noise	
noise	84	52	671	noise		noise	
noise	84	58	568.39	not identified		not identified	
noise	84	79	476.33	noise		noise	
Unidentified_22	103	56	710.28	1,2,3,4,6,7,8,8a-Octahydronaphthalene-6,7-diol, 5,8a-dimethyl-3-isopropenyl-, cyclic carbonate, trans-	91	not identified	
Unidentified_23	103	74	783.11	not identified		not identified	
Unidentified_24	103	74	858.86	1,3-Xylyl-15-crown-4, 2-(4-phenyl-1,3,2-dioxaborolan-2-yl)-	91	not identified	

Metabolite name	Quant ion	Second ion	Retention time (sec)	PBM top hit	PBM %match	NIST top hit	NIST %match
Unidentified_34	103	74	735.2	cis-Resveratrol, 3TMS	91	not identified	
Unidentified_25	103	75	750.68	HEXAKISTRIMETHYLSILYL GLUCITOL-2-D1 ETHER	59	not identified	
Unidentified_35	103	75	795.21	DL-Glyceraldehyde, bis(trimethylsilyl) ether, ethyloxime (isomer 2)	68	not identified	
Unidentified_38	103	83	748.57	Iminodiacetic acid, 3TBDMS derivative	60	not identified	
Unidentified_26	103	84	728.56	1,2,3,4,6,7,8,8a-Octahydronaphthalene-6,7-diol, 5,8a-dimethyl-3-isopropenyl-, cyclic carbonate, trans-	91	not identified	
Unidentified_27	103	98	744.22	not identified		not identified	
Unidentified_36	116	115	688.3	Gluconic acid, 2,3,4,5-tetrakis-O-(trimethylsilyl)-, trimethylsilyl ester, bis(trimethylsilyl) phosphate, D- (CAS) $$ 6-PHOSPHOGLUCONIC ACID-HEPTATMS $$ O-HEPTAKIS(TRIMETHYLSILYL)-D-GLUCONATE-6-PHOSPHATE	59	not identified	
Unidentified_1	116	75	511.6	L-Alanine, N-(trimethylsilyl)-, trimethylsilyl ester (CAS) $$ ALANINE-DITMS $$ N,O-DI-TMS-.ALPHA.-ALANINE $$ TRIMETHYLSILYL ESTER OF N-TRIMETHYLSILYL-L-ALANINE $$ Alanine, N-(trimethylsilyl)-, trimethylsilyl ester, L-	72	not identified	
Unidentified_2	117	103	754.84	3-Hydroxy-12-ketobisnorcholanic acid	91	not identified	
Unidentified_3	128	100	624.63	(4E)-2,3-BENZO-2,4-CYCLOOCTADIENOL	47	not identified	
Unidentified_4	128	75	629.27	1-FLUOROBICYCLO(2.2.2)OCTANE	45	not identified	
Unidentified_41	133	59	431.19	N,O,O-tri(trimethylsilyl)-1-c(13)-N-carboxy-glycine	60	not identified	
Unidentified_5	155	103	558	not identified		not identified	
Unidentified_30	188	172	447.81	6,7-Dimethyl-1,2,3,4-tetrahydrodibenzothiophene $$ Dibenzothiophene, 1,2,3,4-tetrahydro-6,7-dimethyl- (CAS)	64	Phenol, 2,6-bis(1,1-dimethylethyl)-4-isocyano-	41.2
Unidentified_31	205	117	608.37	Acetic acid, 2,2'-oxybis-, bis(trimethylsilyl) ester (CAS) $$ DIGLYCOLIC ACID-DITMS $$ BISTRIMETHYLSILYL DIGLYCOLIC ACID	50	no hit	

Metabolite name	Quant ion	Second ion	Retention time (sec)	PBM top hit	PBM %match	NIST top hit	NIST %match
Unidentified sugar 1 (monosaccharide)	217	204	665.78	.beta.-D-Galactofuranose, 1,2,3,5,6-pentakis-O-(trimethylsilyl)- (CAS) $$ TRIMETHYLSILYL 2,3,5,6-TETRA-O-TRIMETHYLSILYL-.BETA.-D-GALACTOFURANOSIDE $$ Galactofuranose, 1,2,3,5,6-pentakis-O-(trimethylsilyl)-, .beta.-D-	59	not identified	
Unidentified_33	220	218	561.26	Butanedioic acid, bis(trimethylsilyl) ester (CAS) $$ DI-TMS SUCCINATE $$ SUCCINIC ACID-DITMS $$ bis-TMS butanedioate $$ 1,4-butan-dioic acid bis(trimethylsilyl) ester	52	not identified	
Unidentified_6	221	55	489.78	not identified		not identified	
Unidentified_7	241	148	558.97	MONOMETHYLPHOSPHATE 2TMS	43	not identified	
Unidentified_42	241	84	442.26	(3RS,3aRS,7RS)-7-Allyl-3-phenyl-3,3a,4,5,6,7-hexahydro-benz[c]isoxazole $$ 2,1-Benzisoxazole, 3,3a,4,5,6,7-hexahydro-3-phenyl-7-(2-propenyl)-, (3.alpha.,3a.alpha.,7.beta.)-	64	Phosphoric acid, bis(trimethylsilyl)monomethyl ester	85.3
Unidentified_8	243	129	717	not identified		not identified	
Unidentified_9	258	116	423.53	not identified		not identified	
Unidentified_10	267	75	619.63	not identified		not identified	
Unidentified_11	272	200	672.4	[865] 2,6-diaminopimelic acid 2 [18.304]	83	not identified	
Unidentified_12	281	148	435.69	Propanedioic acid, bis(trimethylsilyl) ester (CAS) $$ BISTRIMETHYLSILYL MALONIC ACID $$ MALONIC ACID 2TMS $$ Malonic acid, bis(trimethylsilyl) ester $$ propan-di-oic acid bis(trimethylsilyl) ester $$ Silanol, trimethyl-, malonate (2:1) $$ Bis(trimethylsil	59	Pentasiloxane, dodecamethyl-	41.2
Unidentified_13	283	75	712.26	.alpha.-D-Galactopyranoside, methyl 2,3-bis-O-(trimethylsilyl)-, cyclic butylboronate (CAS) $$ A-GALACTOPYRANOSIDE-1-METHYL-4,6-BUTYLBORONATE-2,3-DITMS	46	noise	

**Metabolite name**	**Quant ion**	**Second ion**	**Retention time (sec)**	**PBM top hit**	**PBM %match**	**NIST top hit**	**NIST %match**
Unidentified sugar 3 (disaccharide)	319	204	834.1	[4623424] sophorose 2 [25.189]	83	2-_-Mannobiose, octakis(trimethylsilyl) ether, methyloxime	54.8
Unidentified_37	324	245	832.74	[6802] guanosine 2 [24.847]	68	Guanosine, penta(trimethylsilyl)-	58.6
Unidentified_14	356	299	635.8	3-Methyl-3-(benzo[b]thiophene-3-yl)-1H,3H-benzo[c]furan-1-one $$ 1(3H)-Isobenzofuranone, 3-benzo[b]thien-2-yl-3-methyl- (CAS)	64	not identified	
Unidentified_39	361	204	848.89	[439193] isomaltose 2 [25.863]	83	not identified	
Unidentified_15	376	204	726.21	not identified		not identified	
Unidentified_43	387	204	791.3	BIS(DIFLUOROPHENYLSILYL-METHYLAMINO)-DIMETHYLSILANE $$ Silanediamine, N,N'-bis(difluorophenylsilyl)-N,N',1,1-tetramethyl- (CAS)	64	not identified	
Unidentified_16	398	229	612.9	Putrescine, 4TMS derivative	74	noise	
Unidentified_40	405	318	766.16	L-MYO-INOSITOL-2-PHOSPHATE-HEPTATMS $$ myo-Inositol, 1,3,4,5,6-pentakis-O-(trimethylsilyl)-, bis(trimethylsilyl) phosphate (CAS) $$ HEPTAKIS(TRIMETHYLSILYL)MYO-INOSITOL-2-PHOSPHATE $$ Inositol, 1,3,4,5,6-pentakis-O-(trimethylsilyl)-, bis(trimethylsilyl) p	50	D-Myo-Inositol, 1,2,4,5,6-pentakis-O-(trimethylsilyl)-, bis(trimethylsilyl) phosphate	33.6
Unidentified_29	416	103	809.15	4-Estren-3,17-dione, di-trimethylsilyl	64	19-Norethandrolone tbdms	38.5
Unidentified_17	440	171	725.6	Benzo[e]naphth[2',1':4,5]indeno[1,2-b]indol-2-ol, 1,2,3,4,4a,4b,5,6,6a,7,14,14a,14b,15,16,16a-hexadecahydro-4a,6a-dimethyl-, acetate (ester), [2S-(2.alpha.,4a.alpha.,4b.beta.,6a.alpha.,14a.beta.,14b.alpha.,16a.beta.)]-	64	Benzo[e]naphth[2',1':4, 5]indeno[1,2-b]indol-2-ol, 1,2,3,4,4a,4b,5,6,6a,7,14, 14a,14b,15,16,16a-hexadecahydro-	86.8
Unidentified_18	56	55	715.99	not identified		not identified	

Metabolite name	Quant ion	Second ion	Retention time (sec)	PBM top hit	PBM %match	NIST top hit	NIST %match
Unidentified_19	70	55	762.34	4-Chloro-N-(4-chloro-2-phenoxyphenyl)benzenesulfonamide, TMS derivative	95	not identified	
Unidentified sugar 2 (disaccharide)	70	58	666.66	.beta.-D-Lactose, (isomer 2), 8TMS derivative	64	not identified	
Unidentified_20	72	55	417.55	not identified		not identified	
Unidentified_28	75	74	451.28	[1,1'-Biphenyl]-2-ol (CAS) $$ o-Phenylphenol $$ o-Hydroxydiphenyl $$ 1-HYDROXY-2-PHENYLBENZENE $$ ORTHO-PHENYLPHENOL $$ 2-Hydroxybiphenyl $$ 2-HYDROXY-BIPHENYL $$ o-Xenol $$ Remol TRF $$ Orthoxenol $$ Dowicide 1 $$ Phenylphenol $$ o-Biphenylol $$ o-Diphen	58	2-Oxa-3-azabicyclo[4.4.0]dec-3-ene, 5-methyl-1-trimethylsilyloxy-, N-oxide	42.2
Unidentified_32	75	74	565.92	[51] alpha ketoglutaric acid [13.859]	91	Pentanedioic acid, 2-(methoxyimino)-, bis(trimethylsilyl) ester	95.9
Unidentified_21	84	75	732.6	XYLITOL 5TMS $$ Xylitol, 1,2,3,4,5-pentakis-O-(trimethylsilyl)- (CAS) $$ Trimethylsilyl ether of xylitol $$ XYLITOL-PENTATMS	43	not identified	
DL-Leucine	86	75	437.73	DL-Leucine, N-acetyl-, ethyl ester (CAS) $$ Leucine, N-acetyl-, ethyl ester, DL- $$ N-Acetyl-DL-leucine ethyl ester	59	not identified	
Octahydronaphthalene	103	75	821.21	1,2,3,4,6,7,8,8a-Octahydronaphthalene-6,7-diol, 5,8a-dimethyl-3-isopropenyl-, cyclic carbonate, trans-	91	not identified	
Palmitic acid	117	75	690.13	Palmitic Acid, TMS derivative	98	Hexadecanoic acid, trimethylsilyl ester	72.5
Pantothenic acid	103	75	676.9	[6613] pantothenic acid 2 [18.371]	72	Pantothenic acid tritms	45.1
Pentanedioic acid	129	75	564.55	Pentanedioic acid, 2-[(trimethylsilyl)oxy]-, bis(trimethylsilyl) ester	83	Pentanedioic acid, 2-[(trimethylsilyl)oxy]-, bis(trimethylsilyl) ester	97.6

**Metabolite name**	**Quant ion**	**Second ion**	**Retention time (sec)**	**PBM top hit**	**PBM %match**	**NIST top hit**	**NIST %match**
Phenylalanine	218	192	584.99	PHENYLALANINE 2TMS $$ TRIMETHYLSILYL ESTER OF N-TRIMETHYLSILYL-PHENYLALANINE	91	N,O-Bis-(trimethylsilyl)phenylalanine	54.5
Phenylalanine	229	99	584.33	PHENYLALANINE 2TMS $$ TRIMETHYLSILYL ESTER OF N-TRIMETHYLSILYL-PHENYLALANINE	86	N,O-Bis-(trimethylsilyl)phenylalanine	75.8
Phosphoric acid	299	158	471.54	[1004] phosphoric acid [9.966]	72	Silanol, trimethyl-, phosphate (3:1)	95.2
Putrescine	174	59	613.83	Putrescine	86		
Pyroglutamic acid	156	75	555.36	Pyroglutamic acid	86		
scyllo-Inositol	318	217	682.43	scyllo-Inositol	76		
Stearic acid	117	75	733.85	Stearic acid, TMS derivative	90	Octadecanoic acid, trimethylsilyl ester	48.3
Succinic acid	75	55	486.42	[1110] succinic acid [10.509]	91	Butanedioic acid, bis(trimethylsilyl) ester	90.1
Trehalose	361	191	826.18	Trehalose	91		
Uracil	241	99	495.15	[1174] uracil [10.777]	91	Pyrimidine, 2,4-bis[(trimethylsilyl)oxy]-	76.3
Urea	114	89	467.53	[1176] urea [9.599]	90	Urea, N,N'-bis(trimethylsilyl)-	90
Uric acid	440	74	714.35	Uric acid, N,O,O',O''-tetrakis(trimethylsilyl)-	70	not identified	
Uric acid	441	382	701.5	1H-Purine-2,6,8(3H)-trione, 4,9-dihydro-1,3,4,9-tetrakis(trimethylsilyl)- (CAS) $$ URIC ACID TETRA TMS $$ URIC ACID, O2,O6,7,9-TETRAKIS(TRIMETHYLSILYL)	96	Uric acid, N,O,O',O''-tetrakis(trimethylsilyl)-	85.3
Uric acid	441	382	699.82	[1175] uric acid 1 [19.331]	90	Uric acid, N,O,O',O''-tetrakis(trimethylsilyl)-	88.6

Metabolite name	Quant ion	Second ion	Retention time (sec)	PBM top hit	PBM %match	NIST top hit	NIST %match
Xanthurenic acid	406	74	739.11	[5699] 4,8-dihydroxyquinoline-2-carboxylic acid (xanthurenic acid) [20.829]	91	2-Quinolinecarboxylic acid, 4,8-bis[(trimethylsilyl)oxy]-, trimethylsilyl ester	97.1

Table S7. Amine containing metabolites measured with LC-MS, including multiple reaction monitoring (MRM) range and retention time (RT)

**Metabolite**	**MRM**	**RT (min)**
[13C]3-L-Alanine [M+Amq+H]+	263.1 -> 171.1	5.192
[13C]5[15N]-L-Valine [M+Amq+H]+	294.1 -> 171.1	7.923
[13C]6-L-Leucine [M+Amq+H]+	308.2 -> 171.1	8.245
2-Aminobutyric acid [M+Amq+H]+	274.1 -> 171.1	5.888
3-Hydroxytyramine [M+Amq+H]+	324.1 -> 171.1	6.848
3-Methoxytyramine [M+Amq+H]+	338.1 -> 171.1	8.512
4-Aminophenylacetic acid [M+Amq+H]+	322.1 -> 171.1	8.553
4-Hydroxyproline [M+Amq+H]+	302.1 -> 171.1	8.904
5-Hydroxytryptophan [M+Amq+H]+	391.1 -> 171.1	7.563
Agmatine [M+Amq+H]+	301.2 -> 171.1	4.826
Alanine [M+Amq+H]+	260.1 -> 171.1	5.192
Amino-2-propanol [M+Amq+H]+	246.1 -> 171.1	4.338
Ammonia [M+Amq+H]+	188.1 -> 171.1	2.469
Arginine [M+Amq+H]+	345.2 -> 171.1	2.841
Asparagine [M+Amq+H]+	303.1 -> 171.1	3.633
Aspartate [M+Amq+H]+	304.1 -> 171.1	3.641
Cadaverine [M+2Amq+2H]2+	222.1 -> 171.1	8.027
Citrulline [M+Amq+H]+	346.2 -> 171.1	5.066
Creatinine [M+H]+	114.1 -> 44.0	1.636
Cystamine [M+2Amq+2H]2+	247.1 -> 171.1	5.056
Cystathionine [M+2Amq+2H]+	563.2 -> 171.1	7.12
Cystathionine [M+2Amq+2H]2+	282.1 -> 171.1	7.117
Cystathionine [M+Amq+H]+	393.1 -> 171.1	7.121
Cysteamine [M+Amq+H]+	248.1 -> 171.1	5.689
Cysteine [M+Amq+H]+	292.1 -> 171.1	5.2
Cystine [M+2Amq+2H]2+	291.1 -> 171.1	4.409
Dihydroxyphenylalanine [M+Amq+H]+	368.1 -> 171.1	7.098
Epinephrine [M+Amq+H]+	354.1 -> 171.1	7.208
Ethanolamine [M+Amq+H]+	232.1 -> 171.1	4.323
Ethylamine [M+Amq+H]+	216.1 -> 171.1	5.906
GABA [M+Amq+H]+	274.1 -> 171.1	5.888
Glucosamine [M+Amq+H]+	350.1 -> 171.1	1.422
Glutamate [M+Amq+H]+	318.1 -> 171.1	5.1
Glutamine [M+Amq+H]+	317.1 -> 171.1	4.284
Glutathione [M+Amq+H]+	478.1 -> 171.1	5.732
Glutathione [M+Amq+H]+	478.1 -> 171.1	5.732
Glycine [M+Amq+H]+	246.1 -> 171.1	4.338
Histidine [M+Amq+H]+	326.1 -> 171.1	2.972
Homoserine [M+Amq+H]+	290.1 -> 171.1	5.286
Indole-3-butyric acid [M+Amq+H]+	374.1 -> 171.1	7.991
Isoleucine [M+Amq+H]+	302.1 -> 171.1	8.904
Kynurenine [M+Amq+H]+	379.1 -> 171.1	8.971

Metabolite	MRM	RT (min)
Lanthionine [M+2Amq+2H]2+	275.1 -> 171.1	6.533
Lanthionine [M+2Amq+2H]+	549.2 -> 171.1	6.52
Lanthionine [M+Amq+H]+	379.1 -> 171.1	6.52
Leucine [M+Amq+H]+	302.1 -> 171.1	8.992
Lysine [M+2Amq+2H]+	487.2 -> 171.1	7.538
Lysine [M+2Amq+2H]2+	244.1 -> 171.1	7.528
Methionine [M+Amq+H]+	320.1 -> 171.1	7.811
Methionine sulfoxide [M+Amq+H]+	336.1 -> 171.1	9.173
Norepinephrine [M+Amq+H]+	340.1 -> 171.1	6.221
Normetanephrine [M+Amq+H]+	354.1 -> 171.1	7.208
Octopamine [M+Amq+H]+	324.1 -> 171.1	6.848
Ornithine [M+2Amq+2H]+	473.2 -> 171.1	7.097
Ornithine [M+2Amq+2H]2+	237.1 -> 171.1	7.095
PABA [M+Amq+H]+	308.1 -> 171.1	8.245
Phenethylamine [M+Amq+H]+	292.1 -> 171.1	9.3
Phenylalanine [M+Amq+H]+	336.1 -> 171.1	9.173
Proline [M+Amq+H]+	286.1 -> 171.1	6.286
Putrescine [M+2Amq+2H]+	429.2 -> 171.1	7.578
Putrescine [M+2Amq+2H]2+	215.1 -> 171.1	7.585
Pyridoxamine [M+Amq+H]+	339.1 -> 171.1	6.197
Serine [M+Amq+H]+	276.1 -> 171.1	4.117
Serotonin [M+2Amq+2H]2+	259.1 -> 171.1	9.193
Serotonin [M+Amq+H]+	347.2 -> 171.1	8.039
Spermidine [M+3Amq+3H]3+	219.4 -> 171.1	8.24
Taurine [M+Amq+H]+	296.1 -> 171.1	4.042
Threonine [M+Amq+H]+	290.1 -> 171.1	5.286
Trypanothione [M+2Amq+2H]2+	532.7 -> 171.1	9.195
Tryptamine [M+Amq+H]+	331.2 -> 171.1	9.584
Tryptophan [M+Amq+H]+	375.1 -> 171.1	9.276
Tyramine [M+Amq+H]+	308.1 -> 171.1	8.245
Tyrosine [M+Amq+H]+	352.1 -> 171.1	7.723
Valine [M+Amq+H]+	288.1 -> 171.1	7.931

References

1. Sumner, L.W.; Amberg, A.; Barrett, D.; Beale, M.H.; Beger, R.; Daykin, C.A.; Fan, T.W.M.; Fiehn, O.; Goodacre, R.; Griffin, J.L., *et al.* Proposed minimum reporting standards for chemical analysis. *Metabolomics* **2007**, *3*, 211-221, 10.1007/s11306-007-0082-2.
2. Bearden, D.; Bundy, J.; Collette, T.; Currie, F.; Davey, M.P.; Field, D.H., N. S.; Hancock, D.; Jones, O.M., N.; Rochfort, S.; Sansone, S., *et al.* CIMR: environmental analysis context - Metabolomics Standards Initiative (MSI). http://msi-workgroups.sourceforge.net/bio-metadata/ (9/11/2017),
